# Porous Structuring of Si Microparticles for Li-Ion Battery Anodes by Urea-Assisted Etching

**DOI:** 10.1021/acsomega.5c12477

**Published:** 2026-02-16

**Authors:** Ali Abo-Hamad, Manisha Phadatare, Daniel Brandell, Maria Hahlin, Jonas Örtegren

**Affiliations:** † Department of Engineering, Mathematics and Science Education (IMD), 6311 Mid Sweden University, Sundsvall SE-851 70, Sweden; ‡ Department of Chemistry - Ångström Laboratory, 8097 Uppsala University, Uppsala SE-751 21, Sweden; § Department of Physics and Astronomy; X-ray Photon Science, Uppsala University, Uppsala, SE-752 37 UPPSALA, Sweden

## Abstract

Silicon-based anodes offer substantially higher theoretical capacities than graphite in lithium-ion batteries, but their practical deployment is hindered by severe volume changes that induce mechanical degradation and unstable interfacial chemistry. While nanoscaling strategies can mitigate these effects, they often suffer from low tapped density, complex synthesis, and limited scalability. Porous silicon microparticles provide a promising alternative by partially accommodating volume expansion while preserving processability and electrode-level integrity. Here, a HF-free urea-assisted etching strategy is employed to generate porous silicon microparticles under mild conditions, leveraging the coupled action of thermally induced structural disruption and chemically driven surface modification. Control experiments confirm that the combined action of these effects is essential to achieve BJH-resolved mesoporosity and increased surface area. The resulting porous silicon exhibits oxygen- and nitrogen-containing surface functionalities. Composite electrodes prepared with nanographite and sodium alginate binder at graphite:silicon:binder ratios of 8:1:1, 7:2:1, and 4.5:4.5:1 demonstrate improved electrochemical behavior. In half-cell testing, electrodes containing 10–20 wt % porous silicon deliver stable redox activity and retain 630–880 mAh g^–1^ after 100 cycles at 0.1 C, with Coulombic efficiencies of 98.8–99.7%, whereas higher silicon loadings lead to rapid capacity decay. Cycling-resolved impedance and differential-capacity analyses reveal the formation of a thicker yet mechanically resilient interphase that stabilizes charge-transfer kinetics, while rate capability tests show 65–74% capacity retention at 2 C.

## Graphical abstract

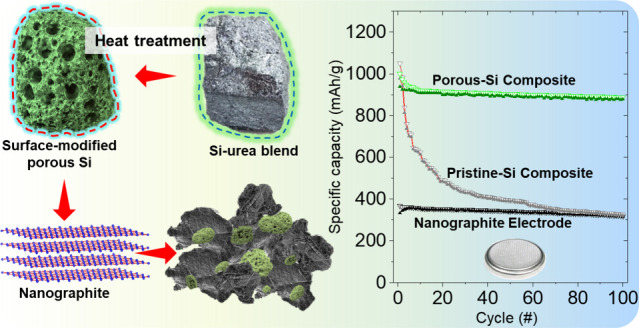

## 1 Introduction

Lithium-ion batteries (LIBs) are indispensable for modern technologies, powering electric vehicles and portable electronics due to their high energy density, long lifespan, and cost efficiency.^1^ However, the growing demand for higher energy densities necessitates alternatives to graphite, which is constrained by a theoretical capacity of 372 mAh g^–1^.^2,3^ Silicon (Si) stands out as a promising anode material, offering a theoretical capacity over ten times greater than graphite. Its natural abundance and low operating voltage (approximately 0.3 V vs Li^+^/Li) further enhance its appeal.^4^

Despite these advantages, the practical application of Si faces significant challenges. Its low electrical conductivity and substantial volume expansion (∼300% during lithiation) result in particle pulverization and undesirable interfacial side reactions, severely compromising cycle stability.^5^ To address these issues, current commercial electrodes incorporate nanosized Si (5–10 wt %),^6,7^ as particles smaller than 150 nm maintain structural stability during cycling.^8,9^ However, nanosizing increases the surface area, exacerbating the solid-electrolyte interphase (SEI) formation, which leads to capacity loss and increased resistance.^10^ Additionally, nano Si production via silane gas condensation is associated with high costs and toxicity concerns.^11^

A scalable alternative involves inducing a porous structure in Si microparticles. Porous architectures have been shown to partially mitigate volume expansion and enhance electrolyte penetration, depending on pore size and connectivity,^12^ thereby facilitating lithium transport within silicon-based anodes.^13^ Several fabrication methods, such as acid etching,^14^ magnesiothermic reduction,^15^ and templating^16^ have enabled the integration of porous silicon into high-performance anodes. For example, Zhou et al.^17^ used a combination of modified metal-assisted chemical etching (MACE) with ball milling to produce nano/microsized porous silicon for LIB anodes, achieving an initial discharge capacity of 2349 mAh g^–1^ at 0.2 C with 95% retention after 100 cycles. Wang et al.^18^ reported a freestanding porous silicon@sulfur-doped porous carbon fiber (Si@SPCF) composite electrode, which retained a reversible capacity of 1113 mAh g^–1^ after 1000 cycles at 2.0 A g^–1^. Li et al.^19^ synthesized porous silicon via magnesiothermic reduction and coated it with polydopamine cross-linked sodium alginate (Si@PDA-SA), yielding an anode with an initial discharge capacity of 3128 mAh g^–1^ and capacity retention of 1382 mAh g^–1^ after 100 cycles. Recently, Ma et al.^20^ demonstrated that overlaying graphite anodes with a porous silicon layer via a sol–gel and etching process notably improved cycling stability (77.5% retention after 500 cycles) and rapid-charging capabilities (65.9% retention at 10C). Despite these advancements, current methodologies frequently involve high temperatures, expensive precursors, complex procedures, prelithiation steps, or hazardous chemicals, thus complicating scalability, increasing production costs, and posing potential environmental and safety concerns.^21^ Therefore, simpler, lower-temperature, and environmentally friendly fabrication methods remain critically needed for the practical advancement of silicon-based anodes.

Building on our recently reported urea-based dual-effect etching strategy,^22^ this study extends this approach to the synthesis of porous silicon microparticles for advanced anode materials. Metallurgical-grade polycrystalline silicon microparticles were selected as the starting feedstock due to their low cost, large-scale availability, and industrial relevance. This silicon grade is widely manufactured for bulk applications and therefore represents a realistic candidate for scalable battery material production. This choice, therefore, aligns with the objective of developing a green and industry-compatible route for silicon-based anodes using safe and inexpensive reagents such as urea. In the proposed dual-effect process, mechanical etching is driven by phase transitions of urea confined within silicon voids, while chemical etching proceeds via ammonia gas generated during urea decomposition at a controlled temperature of 220 °C. The resulting porous silicon microparticles were combined with nanographite and sodium alginate binder to fabricate composite electrodes at nanographite:silicon:binder ratios of 8:1:1, 7:2:1, and 4.5:4.5:1. For comparison, reference electrodes were prepared using pristine silicon composites at the same ratios, as well as a nanographite-only formulation (9:0:1). This type of nanographite was chosen over conventional graphite for its higher surface area, improved electronic conductivity, and proven synergy with silicon in composite anodes, which facilitates both uniform silicon dispersion and enhanced cycling stability.^23–25^ Both prior studies and in-house comparisons demonstrated that nanographite, especially when used with sodium alginate binder, yields greater capacity retention and rate performance than standard graphite materials.^26–28^ Systematic evaluation showed that composite anodes containing 10 or 20 wt % porous silicon maintained high specific capacities and stable cycling performance compared to those with untreated silicon, while higher silicon loadings resulted in rapid capacity loss. The porous silicon composites achieved more than twice the capacity of pure nanographite electrodes after 100 cycles, highlighting the benefits of engineered porosity and interfacial stabilization for silicon-based anodes. By addressing silicon’s intrinsic challenges of volume expansion and structural degradation, this work provides a scalable, HF-free platform toward sustainable high-capacity anodes and establishes a mechanistic basis for further optimization toward practical electrode architectures.

## 2 Experimental Section

### 2.1 Materials and Methods

Metallurgical-grade polycrystalline silicon microparticles (SicoMill grade 2E, batch drum no. 35512, purity 98–99%, average particle size ∼5 μm), sourced from Vesta Si Sweden AB (an SKF Group Company) and typically used for silicon nitride production, were used without additional purification or pretreatment. Urea (ACS grade, ≥99.5%) was purchased from Sigma-Aldrich. Nanographite (NG), comprising graphene, multilayer graphene, and graphite nanoplatelets (platelet thickness <100 nm), was synthesized by large-scale liquid-phase shear exfoliation of expanded graphite according to established procedures.^29–31^ Sodium alginate (S.A.) binder (Fisher Scientific, Technical grade, SLR, Cat. No. 10468800) was selected for its rich content of carboxylic groups, high Young’s modulus, and electrochemical stability, significantly enhancing Coulombic efficiency, specific capacity, and cycling stability.^32,33^ Copper foil (0.025 mm thickness, 99.9% purity) was obtained from Goodfellow Cambridge Ltd., lithium chips (16 mm diameter, 0.6 mm thickness) from Nanografi, electrolyte (1 M LiPF_6_ in ethylene carbonate/diethyl carbonate (EC/DEC), 50/50 v/v) from Sigma-Aldrich, and Celgard 2325 separators (25 μm thickness) from Celgard LLC.

### 2.2 Silicon Treatment

Silicon was treated according to a recently reported procedure;^22^ briefly, silicon microparticles were blended with urea in a 1:2 weight ratio (1 g Si:2 g urea) using an agate mortar and pestle for 10 min to ensure homogeneity. All equipment used during blending was rinsed sequentially with deionized (DI) water and ethanol, and dried before use. The silicon-urea mixture was then transferred to a 25 mL PTFE-lined autoclave vessel, placed within a programmable Nabertherm furnace (temperature range: 30–3000 °C) to precisely control the thermal conditions. A two-stage heating protocol was employed: initially, the mixture was heated to 135 °C at 5 °C min^–1^ and held for 1 h to fully melt and facilitate urea infiltration into silicon voids. In the second stage, the temperature was increased to 220 °C at 10 °C min^–1^ and maintained for 12 h under autoclave conditions to enhance interaction between decomposition gases and silicon microparticles.

Post-treatment washing involved repeated rinsing with boiling DI water until residual urea or decomposition byproducts (e.g., biuret, cyanuric acid) were no longer detectable by energy-dispersive X-ray spectroscopy (Section 3.1.1), followed by filtration and drying to obtain porous silicon microparticles.

To quantify silicon mass loss during the etching process, samples were weighed before treatment and again after thermal processing and thorough washing. Mass changes were used to determine the etching yield and corresponding rate. All measurements were carried out using a high-precision analytical balance (±0.1 mg), and the procedure was repeated across three independently prepared batches to evaluate process reproducibility.

### 2.3 Electrode Fabrication

Electrodes were prepared to achieve uniform mass loading of approximately 1 mg cm^–2^ over a total area of 280 cm^2^ (total mass 280 mg). Seven electrode formulations were prepared with varying compositions of NG, untreated or treated silicon microparticles, and S.A. binder as detailed below (Table 1):

**Table 1:** Summary of Electrode Compositions and Abbreviations Used Throughout the Study

			**composition**		
**electrode**	NG:Si:binder	**silicon type**	NG (mg)	Si (mg)	S.A. (mg)
NGE	9:0:1	no silicon	252	0	28
PrSi-10/NGE	8:1:1	untreated silicon (pristine)	224	28	28
PrSi-20/NGE	7:2:1	untreated silicon (pristine)	196	56	28
PrSi-45/NGE	4.5:4.5:1	untreated silicon (pristine)	126	126	28
PoSi-10/NGE	8:1:1	treated silicon (porous)	224	28	28
PoSi-20/NGE	7:2:1	treated silicon (porous)	196	56	28
PoSi-45/NGE	4.5:4.5:1	treated silicon (porous)	126	126	28

Electrode slurries were prepared by dispersing dry components in 30 mL DI water using an Ultra-Turrax T25 homogenizer with an S 25 N-10 G shear head at 10,000 rpm for 1 h. The resulting homogeneous slurry was cast onto copper foil, dried at room temperature for 24 h, and punched into 16 mm diameter disks. The punched electrode disks were subsequently dried in a vacuum oven at 80 °C for 12 h to ensure complete removal of residual moisture before cell assembly. Electrodes were assembled into CR2032 coin cells inside an argon-filled glovebox, using lithium metal counter/reference electrodes, separators, and electrolyte.

### 2.4 Materials Characterization

The textural properties of both pristine and urea-treated silicon microparticles were characterized using nitrogen adsorption–desorption isotherms (TriStar II Plus, Micromeritics), with surface area calculated by the Brunauer–Emmett–Teller (BET) method,^34^ and pore size distribution determined using the Barrett–Joyner–Halenda (BJH) model.^35^ Structural and compositional analyses included scanning electron microscopy (SEM, TESCAN MAIA 3 Triglav) for morphological evaluation and energy-dispersive X-ray spectroscopy (EDS) for elemental distribution. X-ray diffraction (XRD) patterns were collected using a PANalytical X’Pert Powder diffractometer (Cu K_α_ radiation, 2θ = 10–80°), while Raman spectra (Horiba XploRA PLUS, 532 nm excitation) were recorded in the range of 100–1100 cm^–1^ to probe bonding and phase composition. Particle size distribution (PSD) was measured via laser diffraction (Mastersizer 3000, Malvern Panalytical) with an automated wet dispersion unit (Hydro MV), using particles predispersed in DI water to ensure uniformity and prevent agglomeration. X-ray photoelectron spectroscopy (XPS) measurements were carried out on a KRATOS Axis Supra+ instrument utilizing an Al Kα (1486.6 eV) X-ray source. Analyses were performed under ultrahigh vacuum, with high-resolution scans collected at a pass energy of 20 eV and survey scans at 80 eV. A charge neutralizer was employed throughout to mitigate surface charging effects. Binding energies were referenced to the O 1s peak of silicon dioxide at 532.8 eV. Spectral analysis was conducted using Igor Pro 9 software, applying linear background correction and fitting peaks with a mixed Gaussian–Lorentzian function. Thermogravimetric analysis (TGA, Netzsch STA 449 F1 Jupiter) was utilized to probe the thermal stability and evolution of surface functionalities: samples were heated from room temperature to 850 °C in nitrogen (20 °C min^–1^), cooled to 400 °C and held isothermally for 10 min, then ramped from 400 to 1100 °C in oxygen (20 °C min^–1^), with mass changes continuously recorded to track volatilization and oxidation processes. Additional control samples subjected to modified thermal and precursor-access conditions were characterized using the same SEM and nitrogen adsorption protocols to enable direct comparison of porosity evolution.

### 2.5 Electrochemical Measurements

Electrochemical characterization was performed using a PARSTAT MC multichannel potentiostat/galvanostat (Ametek Scientific Instruments) at room temperature. Electrochemical impedance spectroscopy (EIS) was conducted from 0.1 Hz to 100 kHz. Cyclic voltammetry (CV) was carried out in the range of 0.01–1.5 V at a scan rate of 0.1 mV s^–1^. Galvanostatic charge–discharge (GCD) measurements were performed in the voltage window of 0.01–1.5 V, using a constant-current/constant-voltage (CC–CV) charging mode followed by constant-current (CC) discharging, with a nominal rate of 0.1C for cycling stability tests and a range of rates for rate capability evaluation. Specific capacities and current densities were calculated based on the mass of active material (excluding binder).

## 3 Results and Discussion

### 3.1 Materials Analysis

At the outset of the materials analysis, the reproducibility of the urea-assisted silicon etching process was first evaluated. Under identical processing conditions, the treatment consistently resulted in a silicon mass loss of 15.0 ± 0.2 wt %, corresponding to an average etching rate of 12.7 ± 0.3 mg h^–1^, in close agreement with our previous report using the same strategy.^22^ These results indicate that the etching process proceeds in a reproducible manner across independent batches.

Unlike conventional silicon etching routes based on aggressive alkaline or acidic chemistries, which are often optimized to maximize etching rates, the urea-assisted approach employed here is intentionally designed around mild processing conditions, operational safety, and experimental simplicity. The present study does not aim to optimize etching efficiency, porosity degree, or reagent-to-silicon utilization, but rather to establish the feasibility and electrochemical relevance of this approach when applied to silicon microparticles. Further optimization of reagent ratios and processing parameters is therefore beyond the scope of this work and will be addressed in future studies.

The following sections focus on detailed morphological, structural, and surface analyses to elucidate how this etching treatment modifies the silicon particles and to relate these changes to their electrochemical behavior.

#### 3.1.1 Morphology and Elemental Analysis

SEM analysis provided insights into the morphological changes induced by the dual-effect urea-assisted etching treatment. Figure 1 shows representative SEM images of pristine silicon microparticles (PrSi, Figure 1a,b) and porous silicon microparticles obtained after treatment (PoSi, Figure 1c,d).

**Figure 1:**
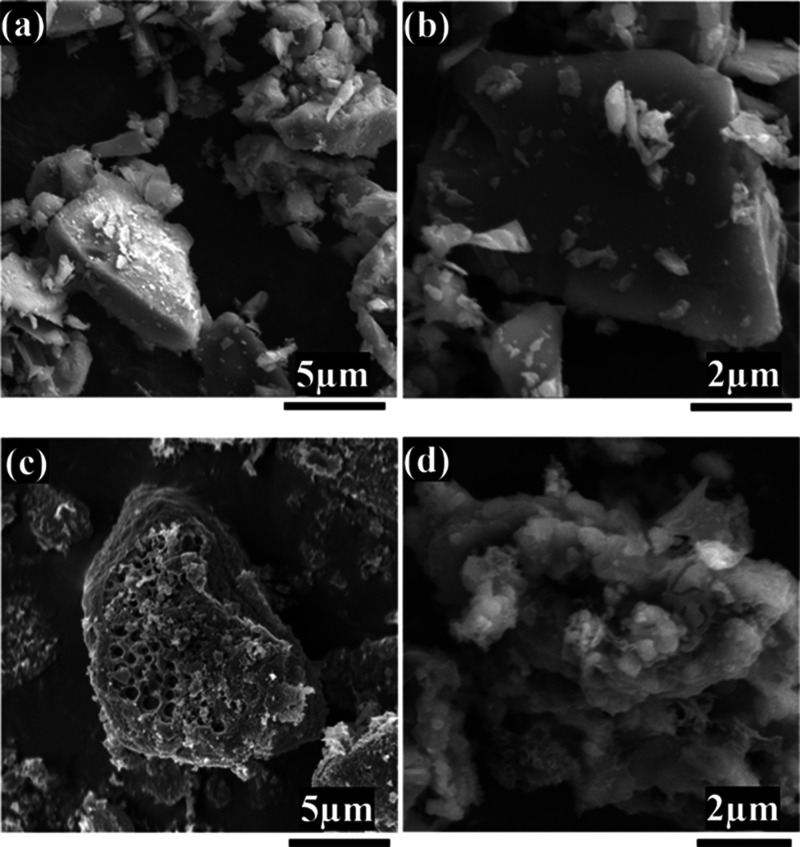
SEM images of pristine silicon (PrSi, a,b) and porous silicon structures generated by urea-assisted etching (PoSi, c,d).

At low magnification, PrSi particles exhibit irregular angular shapes with an average size of approximately 4–5 μm, consistent with the provided manufacturer’s specifications. High-magnification imaging further reveals well-defined edges, smooth surfaces, and a dense, solid internal structure (Figure 1b). Such morphological characteristics confirm the absence of significant porosity or surface roughness in the pristine state, aligning with the expected structural properties of metallurgical-grade silicon intended for industrial processes such as silicon nitride production. The compact morphology observed in PrSi provides mechanical robustness but inherently limits surface area and pore accessibility, which are well-known to restrict lithium-ion diffusion and reduce overall performance in battery applications. In contrast, SEM images of PoSi (Figure 1c,d) clearly show the emergence of pronounced porosity following the urea-assisted thermal treatment. At lower magnification, treated silicon microparticles exhibit significantly disrupted surfaces characterized by distinct open pores and cavities, distributed across particle surfaces. Higher magnification further reveals these features, displaying a high density of fine pore features extending into the silicon structure (Figure 1d). The observed porosity is consistent with the combined influence of mechanical and chemical effects occurring during the thermal decomposition of urea. Mechanically, it is proposed that gaseous byproducts, primarily ammonia (NH_3_) and isocyanic acid (HNCO), generated from urea decomposition may exert localized internal stress within the silicon lattice, causing cracking. Notably, prior mechanistic studies show that as urea and its primary decomposition product, biuret, are heated above 190–210 °C, they form a transient, viscous “foam-like” melt or matrix that expands within confined spaces.^36,37^ As decomposition proceeds, this melt eventually solidifies into intermediate byproducts such as biuret and cyanuric acid.^37,38^ The crystallization and volumetric expansion of these solids within silicon micropores are believed to contribute to further mechanical disruption and structural cracking. Chemically, the reactive gaseous species formed are believed to interact directly with silicon, promoting surface etching and oxidation.^39,40^ This combined mechanism, supported by our morphological observations, aligns with the transformation of the initially dense, nonporous PrSi into a porous structure (PoSi), potentially enhancing electrolyte accessibility and mitigating volumetric changes during electrochemical cycling.

EDS, supported by elemental mapping, was used to examine changes in surface composition resulting from the urea-assisted thermal treatment (Figure 2).

**Figure 2:**
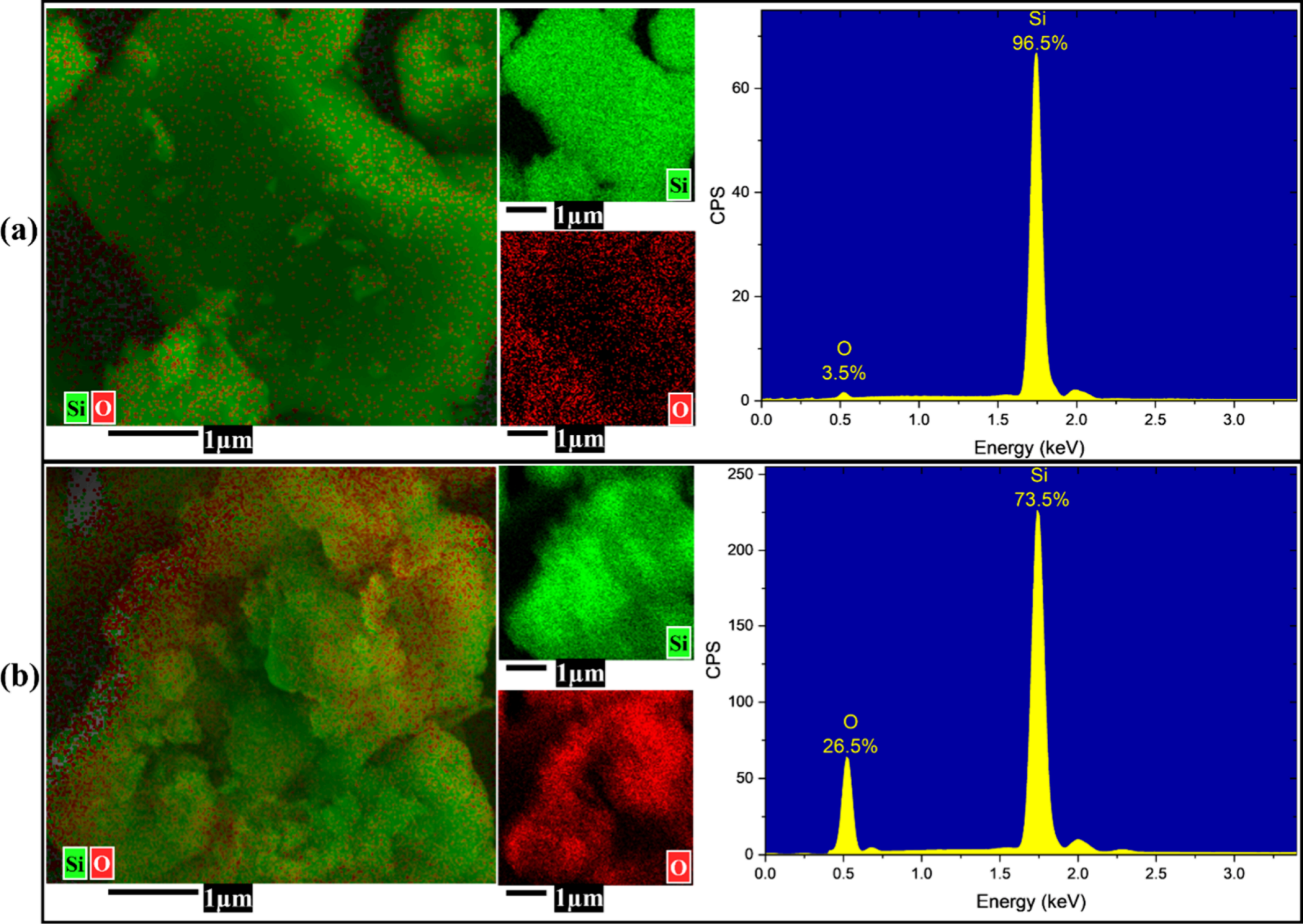
EDS mapping of silicon microparticles. (a) Pristine silicon (PrSi) shows minimal surface oxidation. (b) Porous silicon (PoSi) with significant oxygen incorporation.

In the PrSi (Figure 2a, silicon dominates the spectrum (96.5 wt %), with oxygen content limited to 3.5 wt %, consistent with a native oxide layer typically present on as-received silicon particles. The elemental maps confirm uniform silicon distribution and minimal surface oxygen, in agreement with the dense morphology observed in SEM. In contrast, the treated PoSi (Figure 2b) shows a significant increase in surface oxygen (26.5 wt %) and a corresponding reduction in silicon signal to 73.5 wt %, indicating extensive surface oxidation. This is consistent with the SEM-observed porosity, as the significantly increased surface area in PoSi allows a greater proportion of silicon to undergo surface oxidation, resulting in higher detected oxygen content. Additionally, the confined decomposition of urea at 220 °C under high pressure generates reactive intermediates, primarily ammonia (NH_3_) and isocyanic acid (HNCO), which may alter the Si surface chemistry. While ambient oxygen and residual moisture are primarily responsible for the oxidation of silicon,^41^ NH_3_ and HNCO are believed to further influence the evolution of the fresh surface layer. Moreover, NH_3_ may contribute to the subsequent dissolution or transformation of the oxide layer via nucleophilic interaction or complexation,^42–44^ particularly under elevated temperature and confined conditions. This dynamic cycle of oxidation and partial redissolution likely plays a role in developing the observed porosity and contributes to the chemically altered surface composition of PoSi. Furthermore, the presence of ammonia may facilitate the formation of Si–N bonds at the silicon interface,^45,46^ as later evidenced by XPS analysis.

#### 3.1.2 Textural and Pore Size Analysis

Nitrogen adsorption–desorption analysis was performed to evaluate the textural evolution of silicon microparticles before and after thermal treatment. As shown in Figure 3a, PrSi exhibits minimal nitrogen uptake, consistent with a nonporous or macroporous material. In contrast, PoSi displays a distinct Type IV isotherm with an H3-type hysteresis loop, indicative of mesoporous structures with slit-shaped pores, typically formed from aggregated or layered particles.^47^

**Figure 3:**
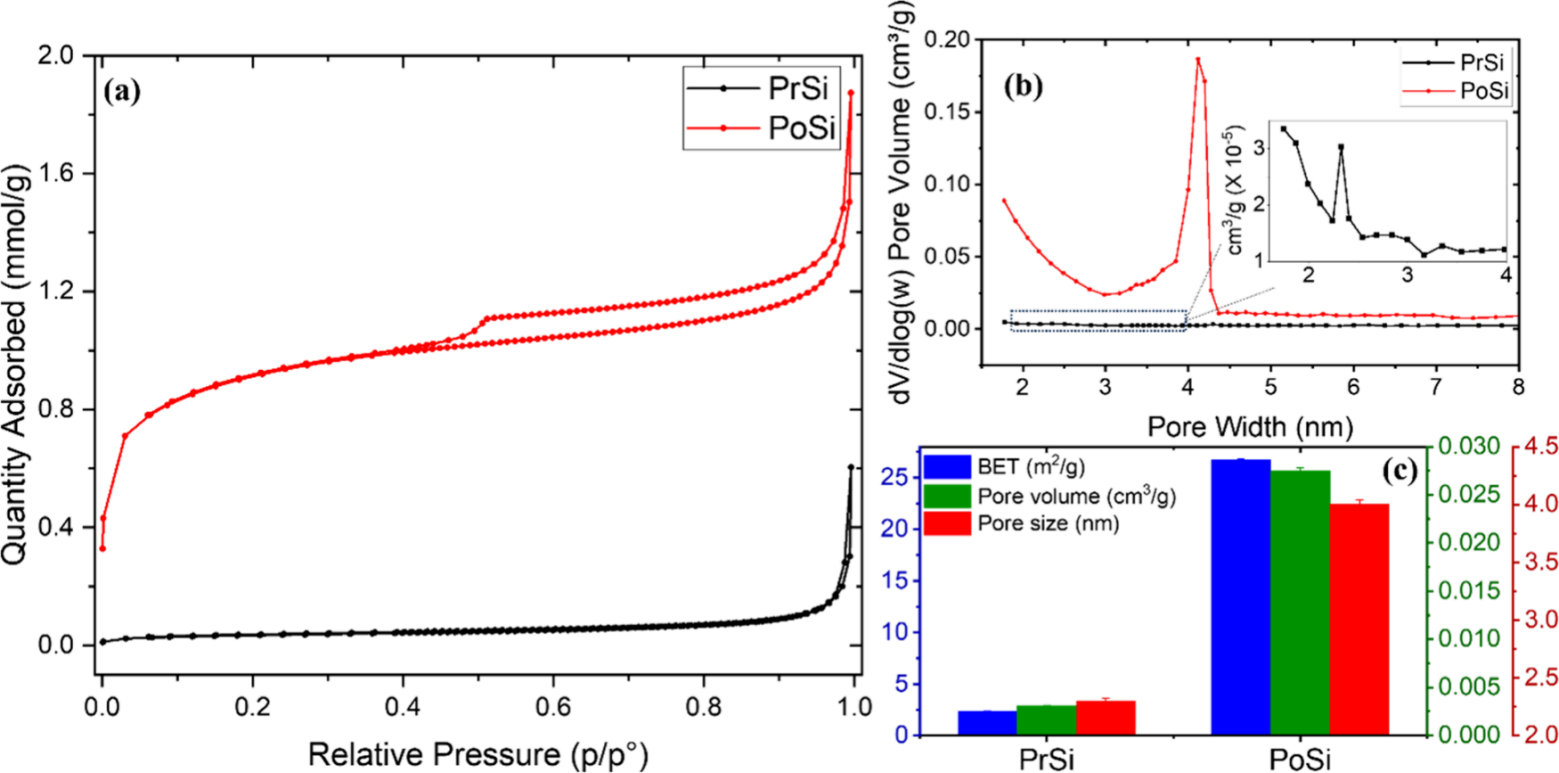
Nitrogen sorption analysis of pristine (PrSi) and porous silicon (PoSi). (a) Adsorption–desorption isotherms, (b) BJH pore size distribution curves, and (c) comparison of BET surface area, pore volume, and average pore diameter.

The BJH pore size distribution (Figure 3b) confirms the emergence of mesoporosity in PoSi, with a broad distribution centered around 4 nm. PrSi shows only a minor signal around 2 nm (Figure 3b, inset), consistent with its compact, nonporous nature. These differences reflect the impact of urea decomposition on pore formation, consistent with the combined influence of mechanical and chemical effects. Quantitative comparison (Figure 3c) reveals a substantial increase in BET surface area from 2.3 m^2^ g^–1^ (PrSi) to 26.7 m^2^ g^–1^ (PoSi), accompanied by a marked increase in accessible pore volume as estimated from nitrogen uptake. These enhancements in surface area and accessible porosity are expected to improve electrolyte infiltration, lithium-ion diffusion, and partial volume accommodation during battery cycling, thereby contributing to improved electrochemical performance. At this stage, BJH analysis is used to identify characteristic pore-size features, while a more detailed quantification of pore-volume contributions across different formation regimes is addressed through targeted control experiments in the following section. While these observations establish the emergence of mesoporosity upon urea-assisted treatment, they do not by themselves distinguish the relative contributions of thermal exposure, chemically driven etching, and stress-assisted structural disruption.

#### 3.1.3 Control Experiments Validating the Dual-Effect Porosification Mechanism

To validate the origin of porosity generated during urea-assisted silicon treatment and to decouple the roles of thermal exposure, chemically driven surface modification, and stress-assisted structural disruption, a set of targeted control experiments was designed in which individual contributions were selectively minimized while maintaining comparable thermal histories, precursor stoichiometry, and post-treatment handling. This approach enables mechanistic assessment of the porosification pathway beyond the direct PrSi-PoSi comparison.

Three control conditions were investigated alongside PoSi. Control 1 (Only Thermal Effect) consisted of PrSi heated under identical autoclave conditions in the absence of urea, isolating the influence of thermal exposure and handling. Control 2 (Minimum Chemical Effect) employed open-system thermal decomposition of a silicon-urea blend prepared using the same 1:2 Si:urea mass ratio as for PoSi. The mixture was heated in a shallow glass beaker on a hot plate (220 °C) under a fume hood to ensure rapid removal of gaseous decomposition products; the treatment was continued until no further bubbling or visible changes were observed (≈1 h). While this configuration does not eliminate chemical interaction entirely, it is expected to substantially reduce sustained gas–solid contact and confined chemical etching. Control 3 (Minimum Mechanical Effect) was prepared by placing urea at the bottom of the autoclave vessel, separating it from the silicon powder using a centrally positioned glass-fiber filter support, and placing the silicon in a ceramic crucible above the barrier. The assembly was held for the same duration as the PoSi preparation, allowing exposure primarily to volatile decomposition products while minimizing direct contact, packing, and expansion-induced mechanical stress. After treatment, silicon was carefully recovered from the crucible to avoid cross-contamination with residual urea or decomposition byproducts. All samples were washed and dried using identical protocols.

##### 3.1.3.1 Morphological Differentiation of Isolated Contributions

SEM images (Figure 4a–c) show that Control 1 retains the dense, angular morphology characteristic of PrSi, with smooth surfaces and no evidence of pore formation, confirming that thermal exposure alone does not induce measurable porosity. Control 2 (Figure 4d–f) exhibits pronounced surface cracking and large void features, consistent with localized fracture and coarse void formation under conditions where chemical interaction is transient and poorly confined. In contrast, Control 3 (Figure 4g–i) displays more uniformly roughened surfaces and fine-scale textural modification, with substantially fewer macroscopic cracks, consistent with vapor-mediated surface alteration rather than bulk fracture. PoSi (Figure 4j–l) exhibits the most extensive surface disruption and nanoscale void features, indicating that the characteristic porous morphology emerges only when both confined chemical interaction and stress-assisted effects are present.

**Figure 4:**
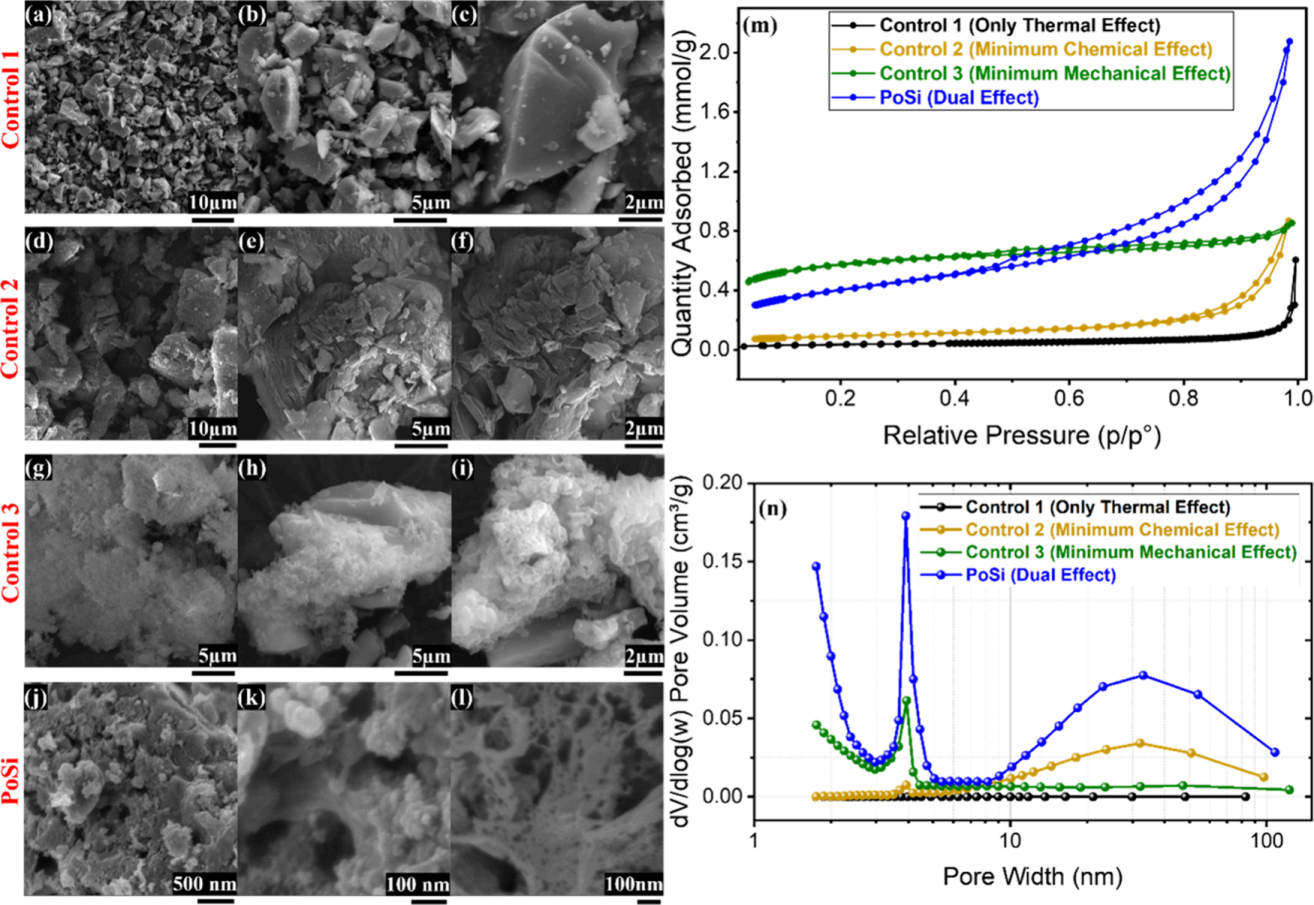
Mechanistic validation of porosity formation in urea-treated silicon using control experiments. Left: SEM images of (a–c) Control 1 (only thermal effect), (d–f) Control 2 (minimum chemical effect; open-system precursor decomposition), (g–i) Control 3 (minimum mechanical effect; vapor-access treatment with physical separation), and (j–l) PoSi (full urea-assisted treatment), illustrating progressive surface modification and pore development. Top right (m): N_2_ adsorption–desorption isotherms showing increasing uptake from Control 1 to PoSi. Bottom right (n): BJH pore-size distributions highlighting distinct pore populations.

##### 3.1.3.2 Nitrogen Sorption Trends and Pore-Volume Metrics

Nitrogen adsorption–desorption isotherms (Figure 4m) corroborate the SEM observations. Control 1 shows negligible nitrogen uptake, whereas progressively higher uptake is observed for Control 2 and Control 3, with PoSi exhibiting the largest adsorption across the full relative pressure range. In these measurements, the pressure-point distribution was adjusted to enhance resolution at higher relative pressures, enabling clearer identification of large-mesopore and void filling that was not accessible in earlier measurements focused on smaller pore sizes. Correspondingly, BET surface areas increase monotonically from 2.6 m^2^ g^–1^ (Control 1) to 4.2 m^2^ g^–1^ (Control 2) and 7.2 m^2^ g^–1^ (Control 3), reaching 26.6 m^2^ g^–1^ for PoSi.

To capture integrated accessible porosity, the total pore volume was calculated from the nitrogen uptake at the highest measured relative pressure (*p*/*p* ^0^)_max_ in each run. This metric reflects the cumulative filling of accessible pores, including large mesopores and voids. In parallel, the mesopore volume (*V*_meso_) was determined by numerical integration of BJH pore-size distributions over the 2–50 nm range, providing a mechanism-sensitive measure of BJH-resolved mesoporosity. Both pore-volume metrics are summarized in Table S.1. An increase in nitrogen uptake at very low relative pressures suggests the presence of pores below ∼2 nm for PoSi and Control 3; however, these features fall outside the reliable resolution of BJH analysis and are therefore discussed qualitatively rather than quantified.

##### 3.1.3.3 BJH Pore-Size Distributions and Mechanism-specific Signatures

BJH pore-size distributions (Figure 4n) provide mechanistically informative distinctions between the samples. Control 1 exhibits an essentially flat distribution, consistent with the absence of mesoporosity. Control 2 shows a bimodal distribution with a modest contribution near ∼4 nm and a pronounced large-mesopore feature centered around ∼30–35 nm, consistent with crack- and void-dominated porosity. Control 3 is dominated by a ∼4 nm mesopore population, with the large-pore contribution strongly suppressed, indicating preferential formation of finer mesoporosity under vapor-access conditions. PoSi combines both features, displaying a dominant ∼4 nm mesopore contribution together with a secondary large-mesopore population in the ∼30–40 nm range.

##### 3.1.3.4 Mechanistic Implication

These results demonstrate that thermal treatment alone does not generate porosity, chemically minimized conditions favor large void formation, and mechanically minimized vapor-access conditions promote mesopore development. Only the fully confined urea-assisted process combines these effects, yielding enhanced surface area and mesopore volume beyond any individual control. These findings provide direct experimental support for a dual-effect porosification mechanism, in which chemically driven surface modification and stress-assisted structural disruption act synergistically under confined conditions.

#### 3.1.4 Structural and Chemical Analysis

The PSD profiles of PrSi and PoSi both exhibit unimodal distributions with sharp peaks, indicating relatively narrow particle size ranges and low polydispersity (Figure 5).

**Figure 5:**
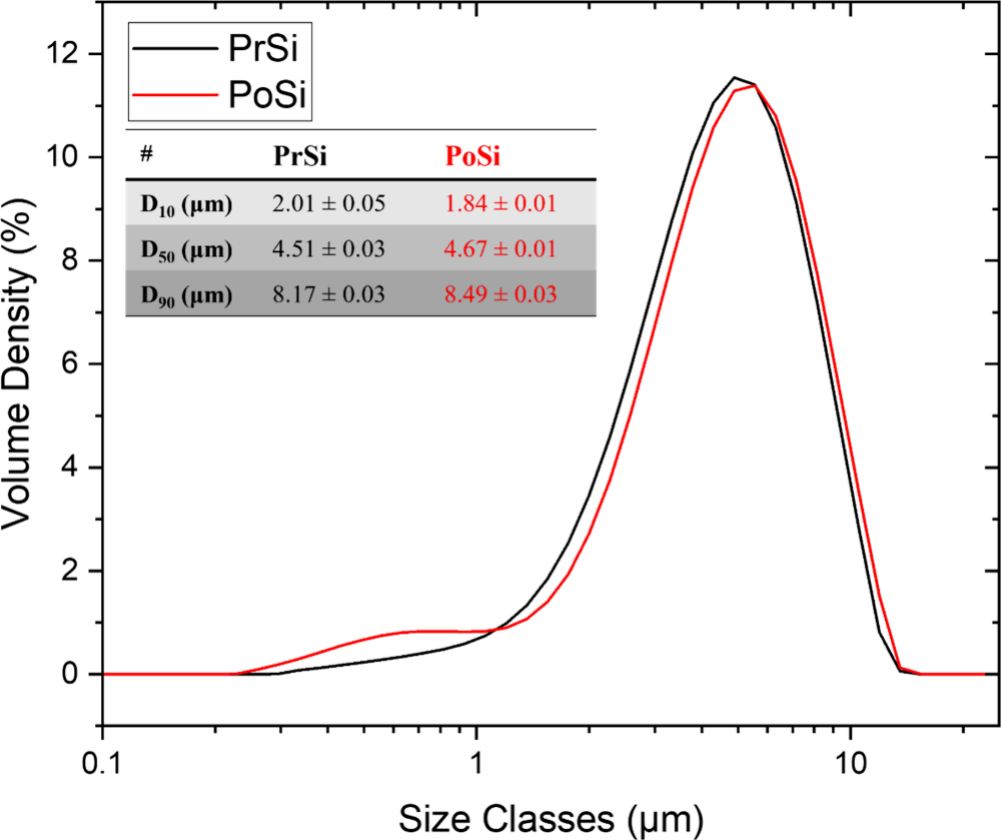
Particle size distribution (PSD) of pristine (PrSi) and porous silicon (PoSi).

Quantitatively, the median particle size (D_50_) and the 90th percentile (D_90_) show only a negligible increase after etching, which may result from minor particle aggregation or changes in particle shape affecting light scattering measurements. In contrast, D_10_ decreases from 2.01 ± 0.05 to 1.84 ± 0.01 μm, indicating potential particle cracking and fragmentation induced by gas evolution and internal stresses during urea decomposition. These observations suggest that, while the surface and internal structure are significantly modified, the bulk dimensions of the microparticles are largely preserved. This structural integrity, combined with enhanced surface area and porosity, is expected to improve ion transport and mechanical resilience during battery cycling.

Raman spectroscopy and X-ray diffraction (XRD) were employed to evaluate changes in the structural order and crystallinity of silicon microparticles before and after urea-assisted etching (Figure 6). Raman spectra (Figure 6a,b) show a prominent peak near 520 cm^–1^ for both PrSi and PoSi samples, corresponding to the first-order transverse optical (TO) phonon mode of crystalline silicon.^48,49^ However, the PoSi sample exhibits an increase in full width at half-maximum (fwhm) from 11.3 to 13.8 cm^–1^ and a small redshift from 520.0 to 518.7 cm^–1^, relative to the PrSi sample. These spectral changes are indicative of lattice distortion and increased disorder introduced during the etching process. The redshift and peak broadening are consistent with phonon confinement effects and the presence of nanostructured domains and surface oxidation, both expected outcomes of pore formation and surface disruption.^50–52^ The absence of amorphous silicon signatures suggests that despite local disorder, the overall crystalline framework is retained.

**Figure 6:**
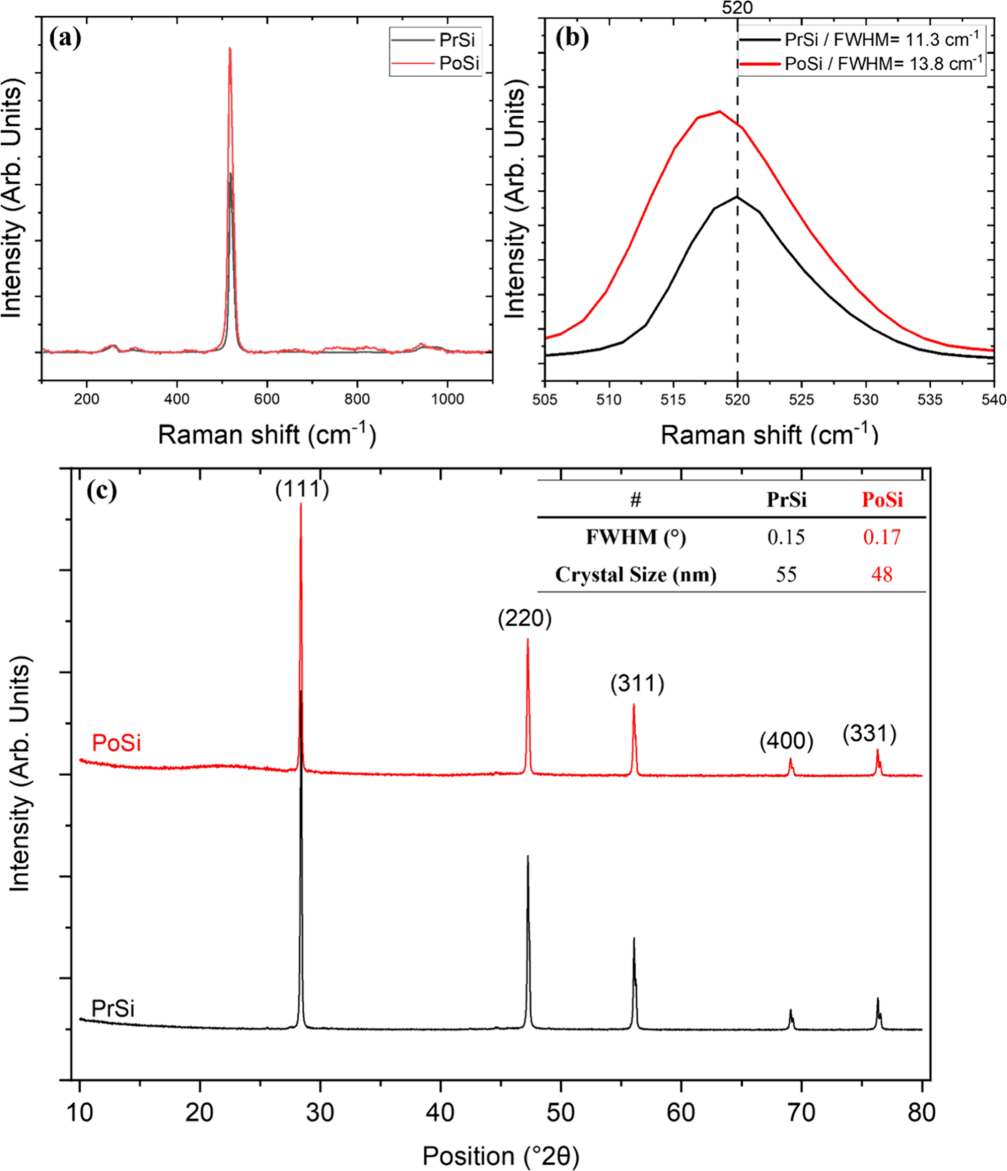
Structural analysis of pristine (PrSi) and porous silicon (PoSi). (a) Raman spectra, (b) corresponding fwhm and peak position comparison, and (c) XRD patterns with an inset highlighting the fwhm of the (111) peaks and calculated crystallite size following the Scherrer equation.

This conclusion is supported by XRD analysis (Figure 6c), where both PrSi and PoSi exhibit identical diffraction patterns, with sharp reflections corresponding to the (111), (220), (311), and higher-order planes of cubic silicon (JCPDS 27–1402), confirming that the crystalline phase remains unchanged after treatment.^53^ A slight broadening of the (111) peak in PoSi, compared to PrSi, indicates a decrease in coherent domain length, likely caused by internal pore formation, partial surface oxidation, and mild particle refinement during thermal etching (reflected in the lower D_10_ value observed in PSD analysis). According to the Scherrer equation,^54^ the broadening corresponds to a crystallite size reduction from 55 nm in PrSi to 48 nm in PoSi. Despite these nanoscale structural modifications, the high overall crystallinity is retained, demonstrating that the treatment alters the surface while preserving the integrity of the bulk silicon lattice.

XPS was used to investigate the surface chemical environment of PrSi and PoSi microparticles (Figure 7). The survey spectra show clear elemental differences following thermal treatment. While both samples contain silicon, oxygen, and carbon, the PoSi spectrum reveals an additional N 1s signal, absent in PrSi, confirming nitrogen incorporation as a result of urea decomposition. In the high-resolution Si 2p spectra, PrSi shows two main features: a peak at ∼99 eV assigned to elemental silicon (Si^0^) and a broader feature near ∼102 eV corresponding to native silicon oxides (SiO_*x*_).^55–58^ In PoSi, the metallic silicon signal is greatly suppressed, and the oxide-related contributions become dominant, suggesting the formation of a substantially thickened oxide layer. This enhanced oxidation is consistent with previous EDS and Raman observations and reflects increased surface reactivity induced by etching. The C 1s spectra for both samples feature a strong peak at ∼285 eV corresponding to hydrocarbon species (C–C/C–H).^58^ In PoSi, the shoulder peaks in the 286–289 eV range become more pronounced, attributed to oxygen-containing functionalities such as C–O and O–C=O.^59^ These species are likely derived from residual urea-based organic fragments adsorbed onto the porous surface during thermal decomposition. No signal is observed near ∼282.5 eV, indicating the absence of silicon carbide (Si–C) formation under the applied treatment conditions.

**Figure 7:**
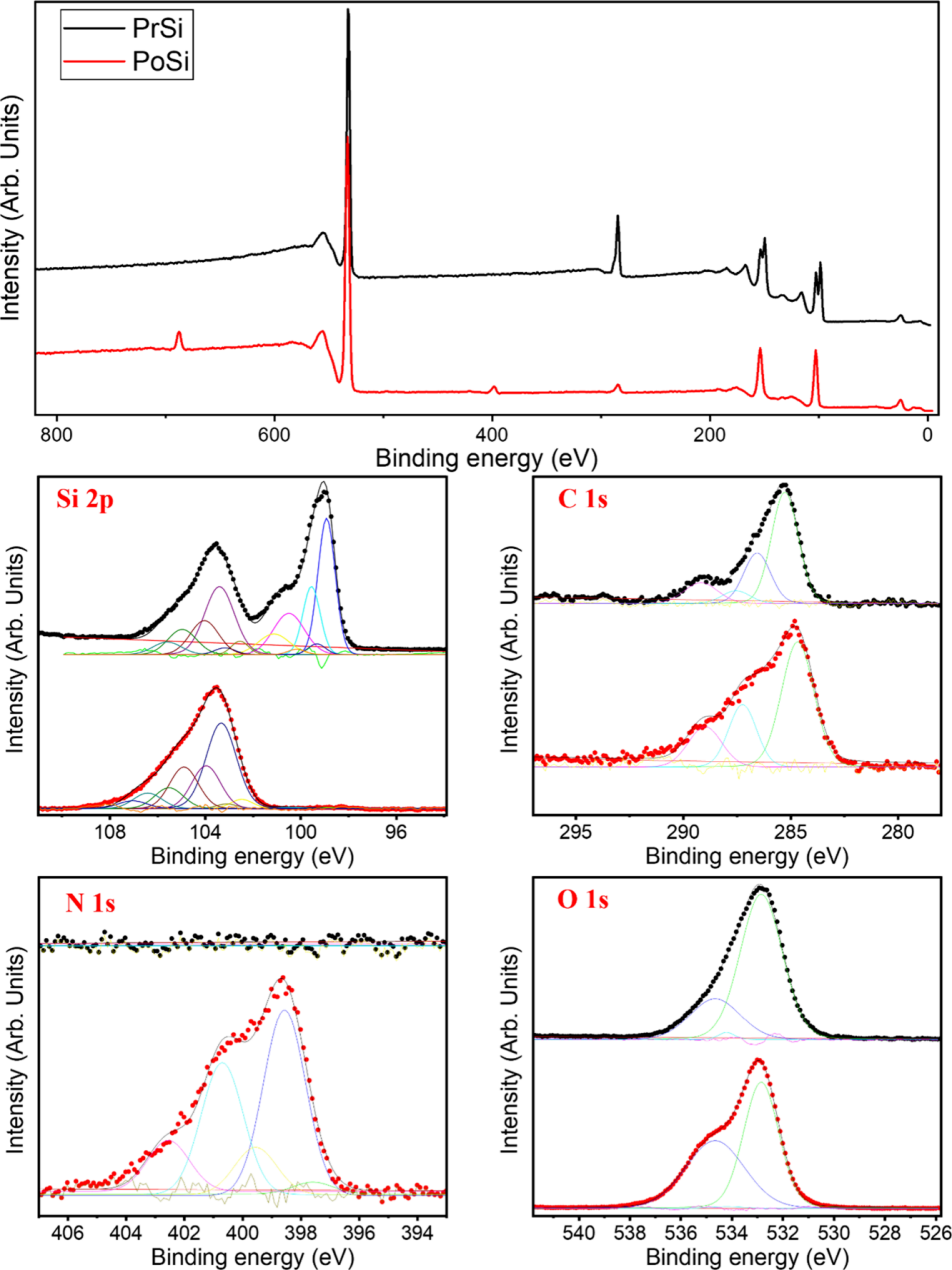
XPS analysis of pristine (PrSi) and porous silicon (PoSi) microparticles.

The N 1s region in PoSi appears as a broad peak spanning 398.5 to 402.5 eV, indicative of nitrogen distributed among several surface bonding environments. Deconvolution identifies four major components: a peak at ∼398.5 eV corresponding to Si–N bonds;^60,61^ features at ∼399.5 eV and ∼401.0 eV assigned to amine (−NH_2_) and protonated amine (−NH_3_^+^) groups, respectively;^62^ and a broader signal at ∼402.5 eV attributed to oxidized nitrogen species (NO_*x*_).^63^ These assignments are based on reference spectra for nitrogen-modified silicon surfaces and are consistent with the thermal breakdown products of urea under the employed treatment conditions. In the O 1s spectra, both samples display a primary peak at ∼532.8 eV, characteristic of Si–O bonding in silicon oxides.^64^ For PoSi, the secondary component near ∼534.6 eV becomes more pronounced and is attributed to surface-adsorbed hydroxyl groups or carbonate-like species.^65^ This further supports the development of a chemically enriched surface with increased functional group density, as a result of the urea-assisted thermal treatment. Quantitative surface composition (atom %) and relative bond contributions derived from XPS peak deconvolution are provided in the Supporting Information (Tables S.2 and S.3).

#### 3.1.5 Thermogravimetric Analysis (TGA)

TGA analysis of PrSi and PoSi microparticles is presented in Figure 8 and Table 2.

**Figure 8:**
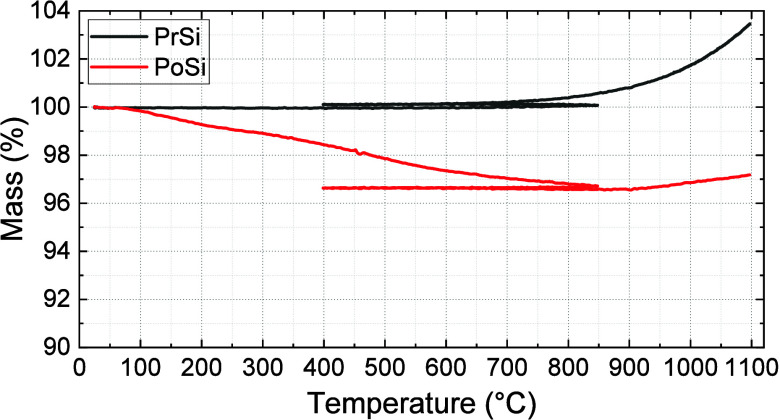
TGA curves of pristine (PrSi) and porous silicon (PoSi) microparticles. Samples were heated from room temperature to 850 °C in nitrogen, cooled to 400 °C, then heated to 1100 °C in oxygen, with mass changes continuously recorded.

**Table 2:** Thermogravimetric Analysis Results for PrSi and PoSi Microparticles

sample	plateau after N_2_ (%)	plateau after air (%)	net mass gain in air (%)	oxidation onset (°C)
**PrSi**	100.1	103.5	3.4	650
**PoSi**	96.7	97.2	0.5	900

Under a nitrogen atmosphere, PrSi exhibited negligible mass loss throughout the heating ramp, reflecting the absence of labile surface groups or volatile components. In contrast, PoSi showed a moderate mass loss of 3.3%, attributed to the volatilization of surface-bound nitrogen- and oxygen-containing species introduced during the urea-assisted etching process, consistent with XPS and EDS analyses. Upon transitioning to air at 400 °C, PrSi underwent rapid oxidation, with mass gain initiating at approximately 650 °C and resulting in a net increase of +3.4% relative to the post-N_2_ plateau. In comparison, PoSi displayed a substantially delayed oxidation onset temperature (∼900 °C) and a much lower overall mass gain (+0.5%), indicating a markedly improved resistance to oxidative mass uptake.

This enhanced oxidation resistance is aligned with the presence of modified surface chemistries generated during confined urea decomposition, including nitrogen- and oxygen-containing species detected by XPS and EDS. To further assess the thermal persistence of these surface functionalities, complementary XPS measurements were performed on PoSi samples subjected to elevated-temperature treatment (800 °C) following the same processing protocol. As summarized in the Supporting Information (Figure S.1 and Table S.3), nitrogen-containing species associated with Si–N bonding environments remain detectable after high-temperature exposure, whereas less thermally stable nitrogen functionalities are substantially diminished. These observations suggest that thermally robust Si–N species may contribute to surface passivation by limiting oxygen access at elevated temperatures. Accordingly, the TGA behavior observed here is consistent with the presence of a chemically modified surface layer that retards oxidation, rather than arising solely from morphological differences between PrSi and PoSi. This anticipated formation of functional surface layers, such as Si–O and Si–N (which are known to act as effective passivating barriers) may offer significant advantages for applications requiring both structural integrity and surface stability.

### 3.2 Electrode Characterization and Electrochemical Performance

#### 3.2.1 Morphology and Composition of Fabricated Electrodes

Figure S.2 shows EDS elemental mapping of the fabricated electrodes, (terminologies and electrode composition as defined in Table 1). The maps illustrate the distribution of carbon (C), oxygen (O), and silicon (Si) across the electrode surfaces, along with corresponding electron images for structural context. The graphite-only reference electrode (NGE) displays strong, uniform C distribution and no Si signal, confirming the absence of silicon. In silicon-containing electrodes (PrSi/NGEs, PoSi/NGEs), a distinct and well-dispersed Si signal is observed, increasing with higher silicon content. This indicates effective integration of both untreated and treated silicon particles into the graphite matrix during slurry preparation and casting. Oxygen is present in all samples due to the inclusion of sodium alginate binder and surface oxides on both silicon and graphite particles. The O signal is homogeneously distributed, suggesting uniform binder coverage across the electrode surfaces. No evidence of elemental segregation or clustering is observed in any electrode. The homogeneous spatial distribution of Si, C, and O confirms that the mixing and fabrication steps produced structurally coherent and compositionally uniform electrodes, suitable for reliable electrochemical characterization.

#### 3.2.2 Electrochemical Behavior: CV

The electrochemical responses of NGE, PrSi-NGEs, and PoSi-NGEs were systematically evaluated by cyclic voltammetry. For clarity, Figure 9 presents fifth-cycle CV curves grouped according to silicon-to-graphite ratio (10:80, 20:70, 45:45), while Supplementary Figure S.3 displays overlays of all five cycles for each electrode.

**Figure 9:**
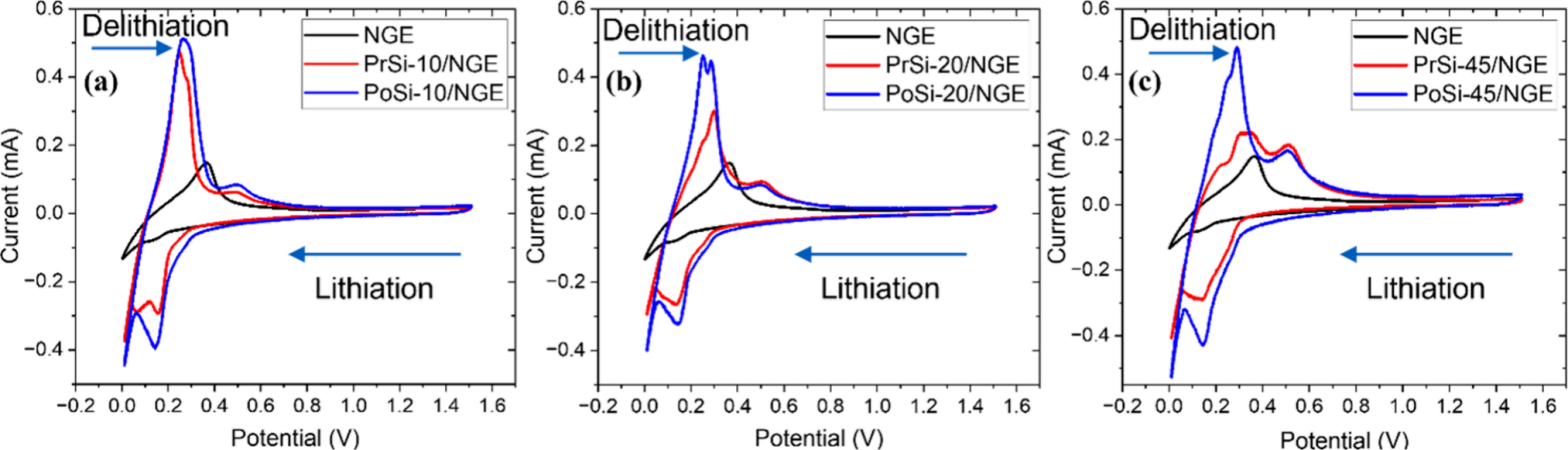
Fifth-cycle cyclic voltammograms of composite electrodes with varying silicon content: (a) 10%, (b) 20%, and (c) 45%, compared to the reference graphite electrode (NGE).

In the 10:80 group (Figure 9a), NGE shows a simple profile with a cathodic peak at ∼0.13 V vs Li^+^/Li (lithiation of graphite to Li_*x*_C_6_) and an anodic peak at ∼0.37 V (delithiation).^66^ Introducing 10% silicon (PrSi-10/NGE) results in additional cathodic peaks at ∼0.16 and ∼0.07 V, assigned primarily to the lithiation of silicon to Li_*x*_Si phases, with possible overlap from deep lithium insertion in graphite at the lowest potential.^67^ In the silicon-containing composites, these cathodic features are consistent with the established room-temperature phase evolution of silicon, in which crystalline Si undergoes electrochemically driven amorphization on first lithiation (c-Si → a-Li_*x*_Si) and, in subsequent cycles, amorphous Si (a-Si) alloys stepwise to Li-poor and Li-rich amorphous phases over ∼0.30–0.10 V, while deep lithiation below ∼0.05 V can thermodynamically enable, but does not necessarily stabilize, the formation of Li-rich crystalline phase (commonly reported as Li_15_Si_4_).^68–70^ On the anodic sweep, new peaks at ∼0.24–0.28 and ∼0.49 V emerge, which are therefore ascribed to staged dealloying of a-Li_*x*_Si and the reformation of predominantly amorphous silicon (a-Si), rather than crystalline Si.^71^ Notably, anodic processes in the ∼0.27–0.30 V range are widely associated with Li extraction from amorphous Li–Si environments containing residual Si–Si networks/clusters, whereas features in the ∼0.43–0.50 V range are commonly linked to late-stage dealloying and can be enhanced when deep lithiation enables transient or spatially confined Li-rich domains rather than bulk crystalline Li_15_Si_4_.^68^ The corresponding porous silicon electrode (PoSi-10/NGE) shows a broader cathodic peak centered around ∼0.14 V, likely reflecting more accessible silicon/NG sites, and a higher, consolidated anodic peak at ∼0.27 V along with a ∼0.49 V feature. Additional insight into the electrochemical processes of the 10:80 composites is provided by differential capacity (d*Q*/d*V*) analysis, calculated from the galvanostatic voltage profiles and presented in Section 3.2.4.2. These profiles are discussed in a subsequent section, where they are used to further corroborate the assignment of silicon-related alloying and dealloying features and their evolution with cycling.

At the 20:70 ratio (Figure 9b), both PrSi-20/NGE and PoSi-20/NGE exhibit more prominent silicon lithiation and delithiation peaks, with PoSi-20/NGE generally presenting slightly higher peak currents, especially at ∼0.14 V (cathodic) and ∼0.25–0.29 V (anodic). While the responses for the two 20% electrodes are similar, the porous sample consistently shows sharper and more well-defined peaks, indicating enhanced electrochemical kinetics and greater utilization of the silicon phase. In the 45:45 group (Figure 9c), both PrSi-45/NGE and PoSi-45/NGE display broad cathodic peaks (∼0.14–0.15 V) and multiple anodic features (PrSi: ∼0.23, 0.34, 0.52 V; PoSi: ∼0.29, 0.50 V), consistent with extensive silicon alloying and dealloying dominating the electrochemical response at high loading.

Looking at the five-cycle overlays (Supplementary Figure S.3), all electrodes initially exhibit a broad cathodic reduction feature spanning approximately ∼0.7–0.3 V in the first cycle, which is characteristic of electrolyte decomposition and SEI formation on graphite- and silicon-containing surfaces. A closer inspection of the first-cycle voltammograms (Figure S.4a–c) reveals systematic differences between PrSi and PoSi composites. In particular, the porous-silicon electrodes display an earlier onset of the broad cathodic reduction current (extending up to ∼0.8 V) and a higher overall current magnitude compared with the corresponding PrSi electrodes at the same silicon loading. This shift to higher onset potentials and increased current intensity is consistent with the larger accessible surface area and higher density of surface oxide and functional groups on PoSi, which promote earlier electrolyte reduction and more distributed interphase formation. Despite this initially enhanced parasitic reduction, the broad SEI-related feature rapidly diminishes after the first cycle for all electrodes, indicating effective passivation of the surface. From the second cycle onward, the silicon-containing electrodes develop stable and reproducible lithiation and delithiation peaks associated with reversible silicon alloying and dealloying processes. Notably, PoSi electrodes (PoSi/NGEs) exhibit sharper, more intense, and more cycle-to-cycle reproducible redox peaks than their PrSi counterparts, reflecting faster reaction kinetics, improved electrochemical accessibility of the silicon phase, and reduced polarization during cycling. These effects are attributed to the engineered porous architecture, which facilitates lithium transport and buffers local stress, together with the presence of stabilizing surface species (Si–O and Si–N) introduced during the urea-assisted treatment.

Additional mechanistic insight into the origin of these differences is provided by first-cycle differential capacity (d*Q*/d*V*) analysis derived from galvanostatic data (Figure S.4d–f). For PoSi-10/NGE, the first discharge d*Q*/d*V* profile shows lithiation features appearing at systematically higher potentials (∼0.19, ∼0.09, and ∼0.07 V) and with higher intensities compared with PrSi-10/NGE (∼0.15, ∼0.07, and ∼0.04 V), indicating earlier activation and a larger fraction of electrochemically accessible silicon. The higher-potential lithiation feature near ∼0.19–0.20 V, which is more pronounced for the porous electrode, is commonly associated with lithiation of oxide-related surface sites and early stage alloying in silicon systems containing native or interfacial Si–O species.^68^ Upon subsequent cycles, the d*Q*/d*V* peak positions for both electrodes stabilize, while their relative intensities diverge: the porous-silicon electrode maintains higher and more stable peak intensities up to 50 cycles, whereas the PrSi electrode shows a progressive attenuation of silicon-related features. This contrast directly corroborates the CV and cycling data, confirming that PoSi enables sustained and kinetically favorable alloying/dealloying pathways, while PrSi undergoes gradual electrochemical deactivation.

#### 3.2.3 Interfacial Properties: EIS

EIS was performed after five CV cycles to evaluate the interfacial resistance components and charge-transfer resistance *R*_CT_ of the composite electrodes (Figure 10). The Nyquist plots were fitted using an equivalent circuit consisting of a solution resistance (*R*), a constant phase element and resistance associated with the solid-electrolyte interphase (CPE_SEI_, *R*_SEI_), a charge-transfer resistance coupled with a double-layer constant phase element (CPE_DL_, *R*_CT_), and a Warburg diffusion element to account for lithium-ion diffusion at low frequencies.

**Figure 10:**
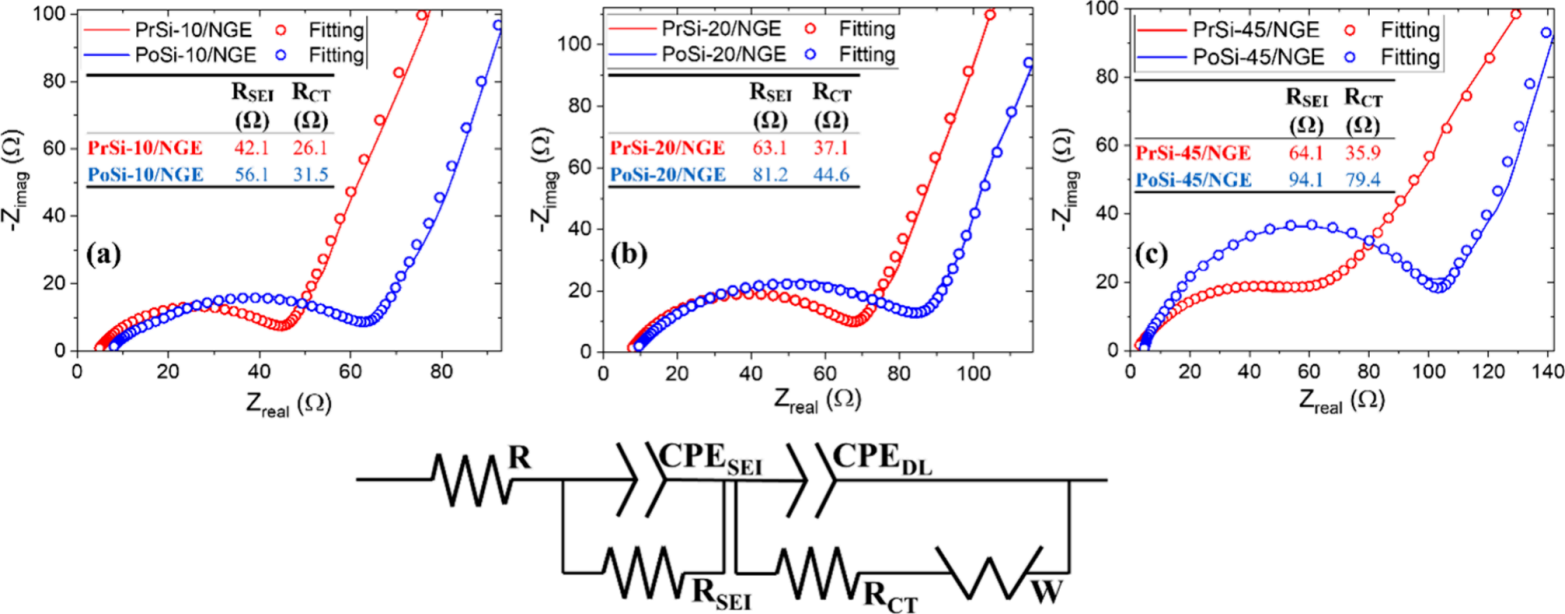
Nyquist plots of representative electrode groups after five CV cycles: (a) 10 wt % Si composites (PrSi-10/NGE, PoSi-10/NGE); (b) 20 wt % Si composites (PrSi-20/NGE, PoSi-20/NGE); (c) 45 wt % Si composites (PrSi-45/NGE, PoSi-45/NGE). Solid lines represent experimental data and symbols denote fitted curves obtained using the equivalent circuit below the plots. Extracted fitting parameters are summarized in Table S.4.

All PrSi/NGE and PoSi/NGE electrodes display a depressed semicircle in the high-to-mid frequency region of the Nyquist plot, which can be deconvoluted into contributions from the SEI resistance (*R*_SEI_) and the charge-transfer resistance (*R*_CT_), followed by a sloped line at lower frequencies corresponding to lithium-ion diffusion. At each silicon loading (10, 20, and 45 wt %), the electrodes containing porous silicon (PoSi/NGEs) exhibit higher *R*_SEI_ and *R*_CT_ values compared to their untreated silicon counterparts (PrSi/NGEs). For instance, at 10 wt % Si, PoSi-10/NGE exhibits an *R*_SEI_ of 56.1 Ω and an *R*_CT_ of 31.5 Ω, compared to 42.1 Ω and 26.1 Ω for PrSi-10/NGE, respectively; analogous increases are observed at 20 and 45 wt % Si (Table S.4).

These higher resistances are attributed to the larger interfacial area and the presence of oxide- and nitride-rich surface species introduced during urea treatment, which promote the formation of a thicker and more passivating interphase. Importantly, this increase in interfacial resistance does not contradict the sharper, well-defined CV features observed for PoSi electrodes. Instead, it reflects a trade-off in which a more resistive but chemically uniform and mechanically robust interphase slightly elevates the initial impedance while enabling more homogeneous lithiation/delithiation and reduced polarization during extended cycling. As the EIS measurements capture the early interphase state after five CV cycles, the superior long-term stability of PoSi/NGEs observed upon prolonged cycling indicates that this initially higher resistance evolves into a stable interface that supports sustained electrochemical reversibility.

#### 3.2.4 Cycling Performance and Rate Capability

##### 3.2.4.1 Long-Term Cycling Stability

The GCD performance of all electrode groups was systematically evaluated over 100 cycles at a current density of 0.1C, as summarized in Figure 11a–c. This cycling protocol (CC–CV charging, and CC discharging within a window of 0.01–1.5 V) was selected to allow near-complete utilization of active material capacity.^72,73^

**Figure 11:**
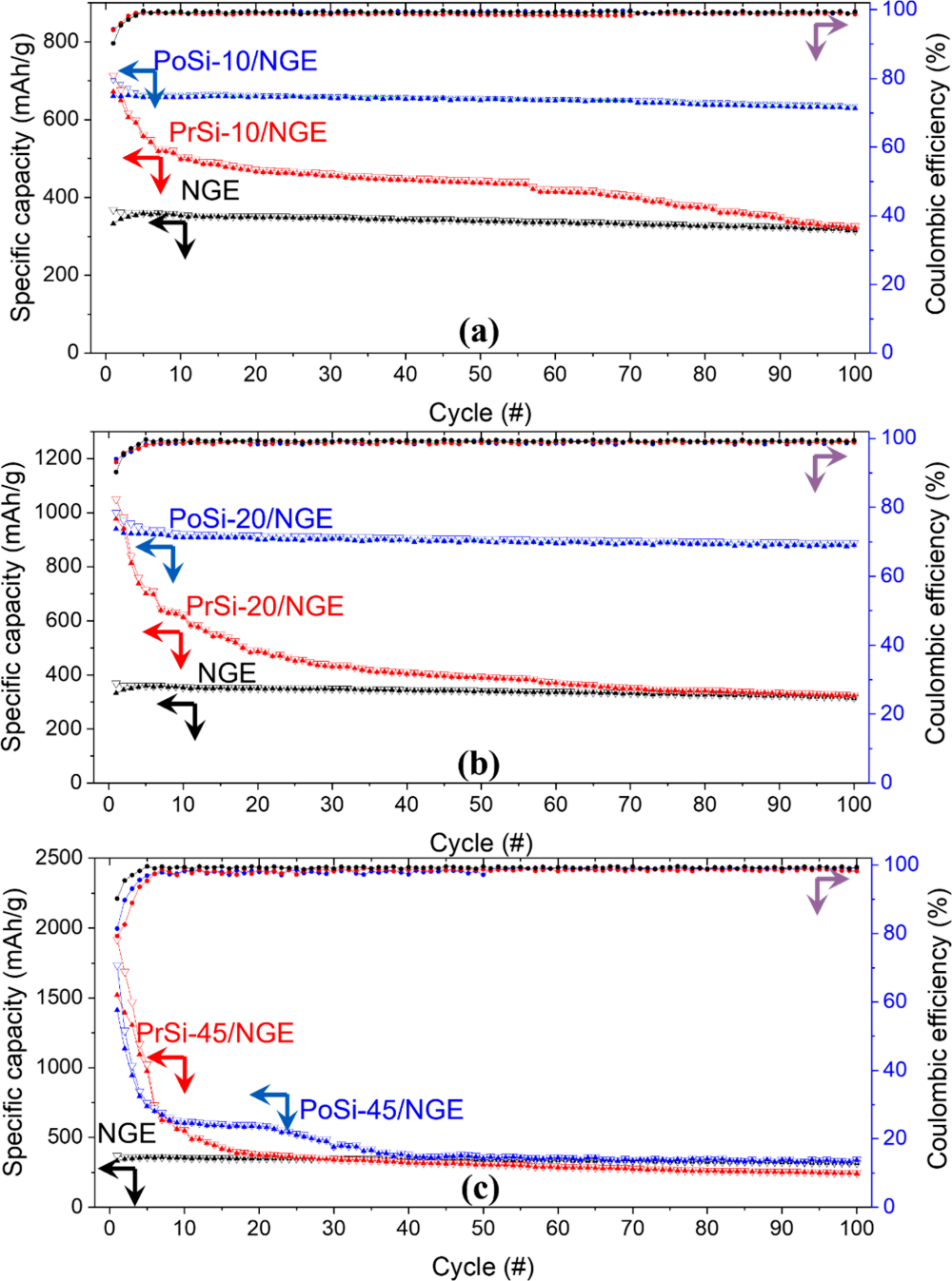
Cycling stability and Coulombic efficiency of graphite and composite electrodes at 0.1C over 100 cycles for (a) 10%, (b) 20%, and (c) 45%, compared to the reference graphite electrode (NGE).

The theoretical capacity of the prepared composite electrodes with a graphite-to-silicon ratio of 10:80 (PrSi-10/NGE and PoSi-10/NGE) was 728 mAh g^–1^ per gram of active material, calculated from the theoretical capacities of graphite (372 mAh g^–1^) and silicon (3579 mAh g^–1^). Similarly, electrodes with ratios of 20:70 (PrSi-20/NGE and PoSi-20/NGE) and 45:45 (PrSi-45/NGE and PoSi-45/NGE) correspond to theoretical capacities of 1084 and 1976 mAh g^–1^, respectively.

The graphite electrode (NGE) delivered a first discharge capacity of 369 mAh g^–1^ and a first charge capacity of 333 mAh g^–1^, corresponding to an initial Coulombic efficiency (ICE) of 90.2%. After a moderate drop during the early cycles, the capacity stabilized and maintained a value of 316 mAh g^–1^ at the 100th cycle, with a total loss of 14%, and with a Coulombic efficiency remaining consistently above 99% beyond the initial cycles, reflecting graphite’s reliable reversibility and minimal side reactions.

Comparing the composite electrodes, Figure 11a highlights the impact of silicon incorporation and structure. PrSi-10/NGE, which integrates pristine silicon with nanographite, exhibited a high initial capacity of 714 mAh g^–1^ (ICE: 94.1%), but this advantage faded rapidly, with capacity dropping to 491 mAh g^–1^ by cycle 20 and further declining to just 326 mAh g^–1^ at cycle 100, corresponding to a 54% total loss from its initial value. In contrast, PoSi-10/NGE, containing porous silicon, achieved a comparable initial capacity of 701 mAh g^–1^ (ICE: 94.3%) but showed much improved stability, retaining 636 mAh g^–1^ after 100 cycles, a total capacity loss of less than 10%. Notably, the per-cycle decay for PoSi-10/NGE averaged below 0.1%, demonstrating the beneficial role of PoSi in sustaining capacity and ensuring robust SEI formation. This stability is consistent with the earlier electrochemical signatures of more distributed initial interphase formation for PoSi electrodes (earlier onset of first-cycle cathodic reduction in CV) and with the higher initial interfacial resistances extracted from EIS (*R*_SEI_ and *R*_CT_; Table S.4), indicating formation of a thicker yet stabilizing interphase that suppresses continuous SEI regrowth during cycling.

Increasing the silicon content, as shown in Figure 11b, further reveals the challenges with PrSi composites. PrSi-20/NGE displayed an initial discharge capacity of 1050 mAh g^–1^ (ICE: 93.1%) but suffered a rapid drop to 623 mAh g^–1^ by the 10th cycle and declined to just 326 mAh g^–1^ by the 100th cycle, a 69% total loss. By contrast, PoSi-20/NGE, which incorporated PoSi at the same loading, started at 1000 mAh g^–1^ (ICE: 94.0%) and retained 888 mAh g^–1^ at cycle 100, reflecting only a 11% decrease over the entire test and an average per-cycle fade also below 0.1%. This improved stability can be attributed to the PoSi architecture, which enhances electrolyte access, facilitates lithium-ion transport, and partially accommodates silicon’s volume changes during cycling. Although the higher surface oxide content of PoSi causes a modest reduction in first-cycle discharge and charge capacities due to additional irreversible lithium consumption, this effect remains well controlled at moderate silicon loadings (10–20 wt %) and does not compromise practical initial Coulombic efficiency. Surface modifications such as Si–O and Si–N species may further contribute to preserving structural integrity and promoting long-lasting silicon utilization within the composite electrode.

The performance at even higher silicon contents is illustrated in Figure 11c. PrSi-45/NGE, with the highest silicon fraction, exhibited an initial capacity of 1919 mAh g^–1^ but suffered major loss within the first few cycles, dropping to 555 mAh g^–1^ by cycle 10 and stabilizing near 247 mAh g^–1^ at cycle 100, corresponding to a loss of nearly 87%. PoSi-45/NGE, started slightly lower (1734 mAh g^–1^, ICE: 81.5%) and demonstrated less severe early capacity fade, retaining 341 mAh g^–1^ after 100 cycles. The reduced ICE at this loading is therefore attributed primarily to the excessive silicon fraction rather than an intrinsic limitation of the porous-silicon design. Although PoSi-45/NGE outperforms the PrSi composite at the same loading, the overall retention remains poor, and the capacity ultimately converged toward the graphite baseline. Further optimization, such as alternative binders, electrode/electrolyte additives, or modified cycling protocols, is required to realize stable cycling in high-Si-based anodes.^74–76^

Overall, these results demonstrate that incorporating PoSi into a graphite matrix enables substantial gains in reversible capacity and cycling stability when the silicon content remains moderate and does not exceed the stabilizing capacity of the graphite framework. At excessive silicon fractions, as in PoSi-45/NGE, the benefits of porosity alone are insufficient to prevent structural degradation and rapid capacity loss, underscoring the critical role of graphite as both a mechanical buffer and a continuous electrochemical host. The pronounced performance gap between pristine- and porous-silicon composites therefore indicates that engineered silicon morphologies are most effective when coupled with appropriate compositional balance, rather than serving as a standalone solution for high-loading silicon anodes. Similar constraints have been reported for silicon-graphite systems employing alternative architectural strategies. For example Ma et al. showed that even nanosized silicon cannot be stably accommodated at high loadings unless supported by graphite matrices with tailored, interconnected porosity, limiting effective silicon content to ∼17 wt %.^77^ In contrast, Yan et al. achieved stable cycling at higher silicon fractions (up to ∼30 wt %) only by combining nanoscale silicon with conformal carbon encapsulation via chemical vapor deposition, thereby maintaining electrical percolation and suppressing interfacial degradation.^78^ Taken together, these comparisons suggest that while porosity and surface chemistry can effectively stabilize moderate silicon loadings (even when starting from micron-scale silicon) higher silicon fractions require additional architectural confinement and protective surface engineering to preserve percolation, limit interphase overgrowth, and sustain long-term electrochemical activity. Although the present study focuses on intrinsic structure–property relationships without external lithium compensation, established strategies such as electrode prelithiation, lithium-containing additives, or advanced SEI-forming electrolyte formulations are widely reported to mitigate first-cycle lithium loss^79,80^ and could be readily integrated in future studies to further improve ICE toward commercially relevant targets.

##### 3.2.4.2 Voltage-Resolved Lithiation and Interfacial Phase Evolution during Cycling

Analysis of the voltage profiles in Figure 12a–c reveals the underlying mechanisms for electrode behaviors. Both PoSi-10/NGE and PoSi-20/NGE display pronounced voltage plateaus at low potentials associated with silicon lithiation and delithiation, superimposed on the typical graphite profiles, and these features persist with minimal polarization over repeated cycles. In the high-silicon electrode, however, these plateaus fade rapidly, reflecting the swift deactivation or isolation of silicon, and explaining the abrupt capacity loss observed in the cycling data.

**Figure 12:**
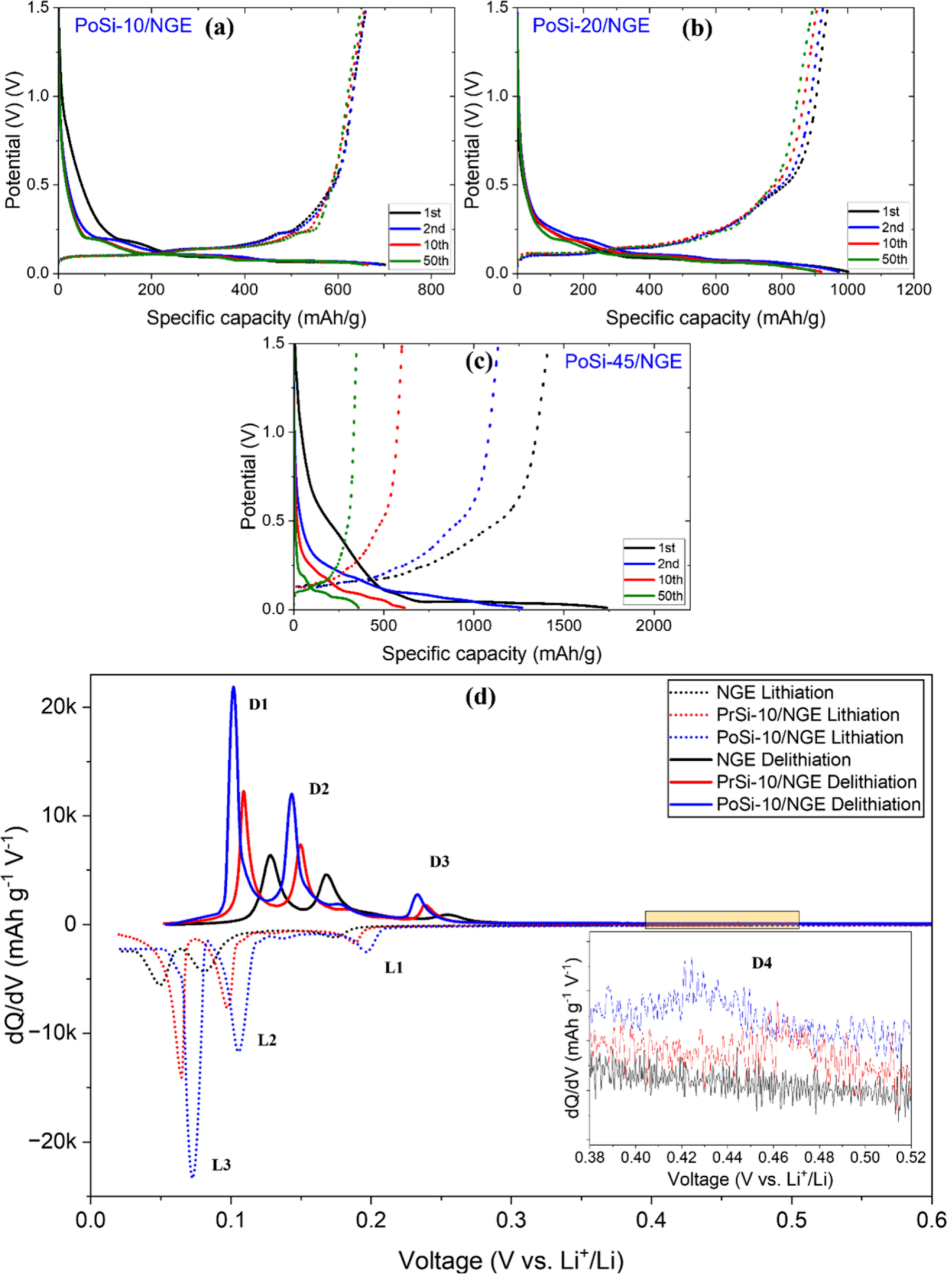
GCD voltage profiles of representative electrodes at selected cycles (1st, 2nd, 10th, and 50th) at 0.1C, highlighting the evolution and stability of lithiation/delithiation plateaus (a–c). Differential capacity (d*Q*/d*V*) profiles at the 50th cycle for NGE, PrSi-10/NGE, and PoSi-10/NGE electrodes, normalized to the total mass of active material, with the inset emphasizing the delithiation region between 0.40 and 0.50 V associated with silicon dealloying (d).

While these voltage profiles clearly differentiate stable and unstable composite formulations, the composite nature of the electrodes and the dominant graphite contribution inherently obscure silicon-specific reaction pathways and interfacial effects. In particular, subtle contributions arising from surface oxide reduction, SEI formation, and silicon phase evolution cannot be unambiguously resolved from the raw galvanostatic curves alone. To address this limitation and to elucidate the voltage-dependent lithiation and delithiation processes of silicon, differential capacity (d*Q*/d*V*) analysis was employed as a complementary, phase-sensitive tool.

The influence of porous-silicon surface chemistry on early electrochemical behavior is first evident in the initial delithiation cycles. As shown in Figure S.4d, the first-cycle d*Q*/d*V* profiles of PoSi-10/NGE exhibit an earlier onset of lithiation and higher peak intensities compared to PrSi-10/NGE, with features emerging near ∼0.19–0.20 V, followed by additional contributions at ∼0.09 and ∼0.07 V. These higher-voltage lithiation features are commonly associated with lithiation of oxide-adjacent silicon environments and partially reduced Si–O species, indicating that the increased surface oxide content of PoSi contributes to additional irreversible lithium consumption and more distributed interphase formation during the first cycle. In contrast, the PrSi composite shows delayed lithiation onset (∼0.15 V) and weaker overall d*Q*/d*V* intensity, consistent with less accessible silicon surface area and a more localized interfacial reaction.

The evolution of these features upon cycling further highlights the role of porosity and surface chemistry in stabilizing the silicon phase. Figure S.4e,f compare the discharge d*Q*/d*V* profiles of PrSi-10/NGE and PoSi-10/NGE over the first, second, 10th, and 50th cycles. For PrSi-10/NGE, the d*Q*/d*V* peak intensities progressively diminish with cycling, despite relatively stable peak positions after the second cycle, likely caused by a gradual loss of electrochemically active silicon due to particle fracture, loss of electrical contact, and repeated SEI rupture and reformation. By contrast, PoSi-10/NGE maintains both peak positions and intensities from the second cycle onward, demonstrating that a larger fraction of silicon remains electrochemically accessible over extended cycling. This behavior is consistent with the formation of a mechanically robust and chemically stable interphase, despite the higher initial interfacial resistance extracted from EIS.

Further insight into long-term silicon utilization is provided by the 50th-cycle d*Q*/d*V* comparison shown in Figure 12d. Across all lithiation and delithiation processes, PoSi-10/NGE consistently exhibits systematic shifts in peak positions relative to PrSi-10/NGE and the graphite-only electrode (NGE). Specifically, lithiation features appear at higher potentials (L1 → L3 at ∼0.20, 0.11, and 0.07 V for PoSi-10/NGE versus ∼0.19, 0.10, and 0.06 V for PrSi-10/NGE), while the corresponding delithiation peaks occur at lower potentials (D1 → D3 at ∼0.10, 0.14, and 0.23 V versus ∼0.11, 0.15, and 0.24 V). These systematic voltage shifts indicate reduced reaction polarization and improved kinetic reversibility in the porous-silicon composite, consistent with its stabilized interfacial resistance upon cycling (as discussed below).

Importantly, examination of the high-voltage delithiation region (D4 at ∼0.40–0.50 V, inset of Figure 12d) reveals a broad, low-intensity feature centered at ∼0.43 V for PoSi-10/NGE, a weaker and less reproducible response near ∼0.45 V for PrSi-10/NGE, and no corresponding feature for NGE. The absence of a sharp peak in this region suggests that large-scale formation and cycling of crystalline Li-rich phases (such as Li_15_Si_4_) are suppressed after extended cycling. Instead, the presence of a broad feature in PoSi-10/NGE is indicative of delithiation from highly lithiated amorphous Li_*x*_Si domains confined within the porous framework. This behavior is desirable, as repeated formation of crystalline Li-rich phases is known to exacerbate mechanical stress and accelerate degradation, whereas stabilized amorphous cycling promotes long-term reversibility.

To further elucidate the origin of the rapid capacity fading observed for PoSi-45/NGE, cycling-dependent electrochemical impedance spectroscopy was conducted after the fifth, 20th, and 50th cycles (Figure 13). While PoSi-10/NGE and PoSi-20/NGE exhibit progressive stabilization or even reduction of both the interfacial resistance (*R*_SEI_) and charge-transfer resistance (*R*_CT_) with cycling, consistent with interphase maturation and preserved electronic percolation, PoSi-45/NGE displays a markedly different impedance evolution. In this high-silicon electrode, R_CT_ increases substantially by the 20th cycle and both *R*_SEI_ and *R*_CT_ rise sharply by the 50th cycle (*R*_SEI_ ≈ 1.05 × 10^3^ Ω and *R*_CT_ ≈ 7.57 × 10^2^ Ω; Figure 13e,f), indicating accelerated interphase thickening and loss of efficient charge-transfer pathways. By contrast, PrSi composite electrodes exhibit a monotonic and much more pronounced increase in both *R*_SEI_ and *R*_CT_ with cycling at all silicon loadings (Figure S.5), reflecting continuous SEI rupture and progressive interfacial degradation rather than interphase stabilization. Interestingly, for the specific case of PoSi-45/NGE, the pronounced impedance growth temporally coincides with the disappearance of silicon-related voltage plateaus and the collapse of reversible capacity toward the graphite baseline, providing direct evidence that excessive silicon loading overwhelms the buffering capability of the porous architecture. The resulting mechanical stress, repeated SEI rupture, and disruption of electronic connectivity lead to progressive electrical isolation of silicon domains (“dead Si”), ultimately dominating the long-term degradation behavior of PoSi-45/NGE.

**Figure 13:**
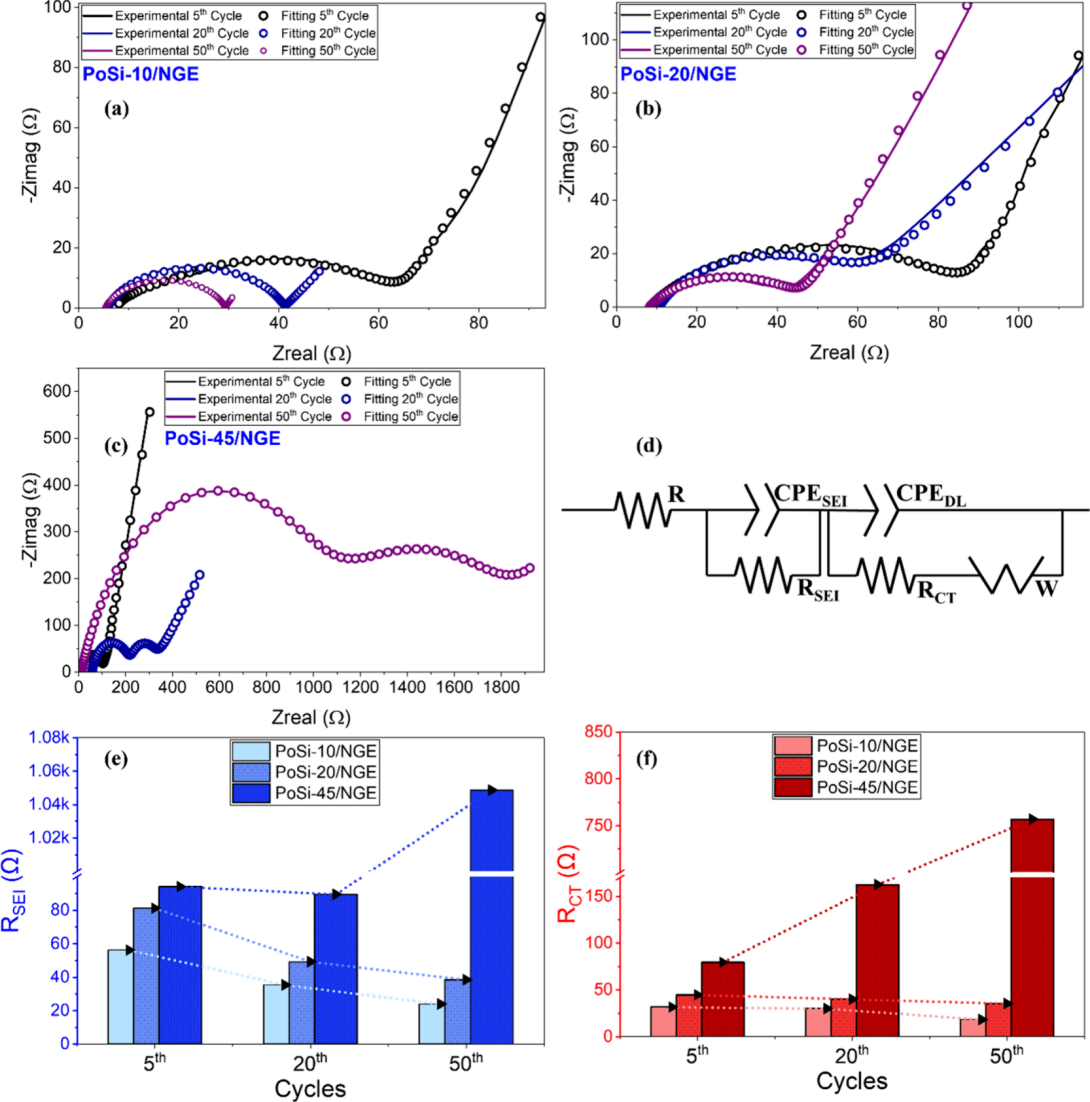
Cycling-dependent impedance evolution of porous-silicon composite electrodes. (a–c) Nyquist plots of PoSi-10/NGE, PoSi-20/NGE, and PoSi-45/NGE measured after the 5th, 20th, and 50th cycles (solid lines: experimental data; symbols: fits). (d) Equivalent circuit used for fitting. (e) Evolution of *R*_SEI_ and (f) *R*_CT_ extracted from the fits as a function of cycle number.

Taken together, the combined voltage-profile, differential capacity (d*Q*/d*V*), and cycling-dependent EIS analyses demonstrate that the increased oxide content and porosity of PoSi electrodes do not impede lithiation, but instead redistribute interfacial reactions during the early cycles and stabilize silicon alloying/dealloying pathways during extended cycling. Although PoSi/NGE exhibits higher initial interfacial resistance and first-cycle irreversibility, these effects promote the formation of a more uniform and mechanically resilient interphase that suppresses polarization growth, preserves silicon-related electrochemical signatures, and maintains stable lithiation plateaus over prolonged cycling. Importantly, this stabilization is corroborated by the evolution of *R*_SEI_ and *R*_CT_ with cycling, which remain moderate or even decrease for PoSi-10/NGE and PoSi-20/NGE, whereas the corresponding PrSi electrodes show continuous impedance growth over 50 cycles (Figure S.5). This voltage- and impedance-resolved perspective provides a mechanistic explanation for the enhanced cycling stability of PoSi-10/NGE and PoSi-20/NGE relative to their PrSi counterparts, while also clarifying the failure mode at excessive silicon loading.

##### 3.2.4.3 Rate Capability and Kinetic Robustness

The rate capability of PoSi-10/NGE and PoSi-20/NGE electrodes is shown in Figure 14 and further corroborates the kinetic advantages of incorporating PoSi into NG-based composites.

**Figure 14:**
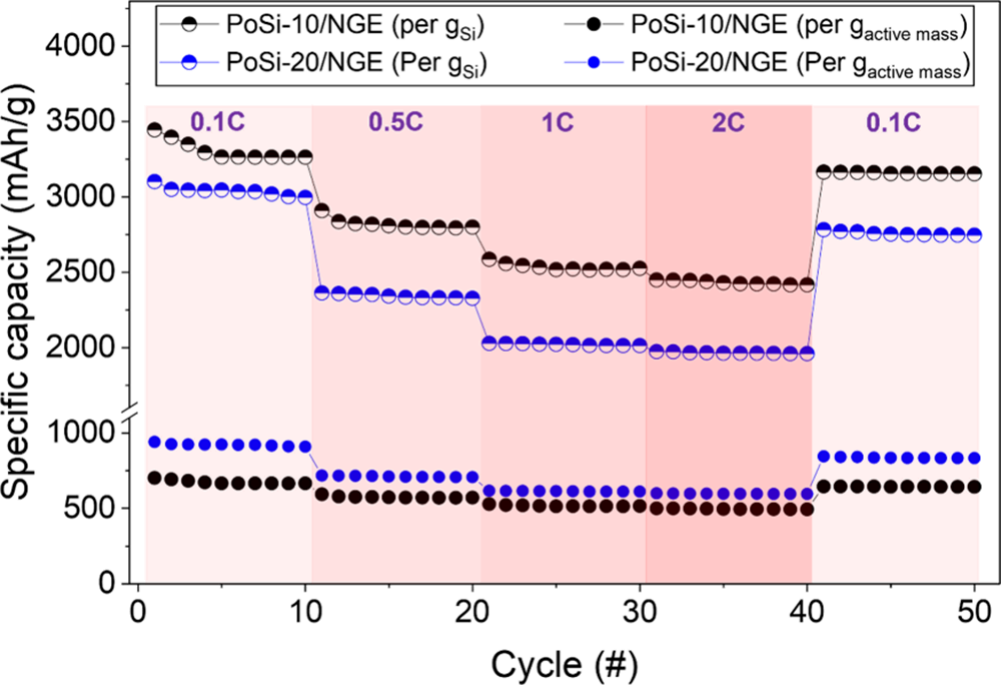
Rate capability of PoSi-10/NGE and PoSi-20/NGE electrodes during stepwise C-rate cycling (0.1C, 0.5C, 1C, 2C, 0.1C; 10 cycles per step). Specific capacities normalized to total active mass (Si + NG) are shown as the lower set of curves, while Si-normalized specific capacities are shown as the upper set of curves.

The rate response is first evaluated on the basis of capacity normalized to total active mass (lower curves in Figure 14), which reflects practical electrode-level performance. As summarized by the 10-cycle block averages in Table S.5, PoSi-20/NGE exhibits higher gravimetric capacity per gram of active material across the full rate window, averaging 920.4 mAh g^–1^_(active)_ at 0.1C (cycles 1–10) and decreasing to 709.8, 612.0, and 595.2 mAh g^–1^_(active)_ at 0.5C, 1C, and 2C, respectively, before recovering to 835.3 mAh g^–1^_(active)_ upon returning to 0.1C (cycles 41–50). PoSi-10/NGE delivers lower absolute capacity per gram of active material, averaging 672.8 mAh g^–1^_(active)_ at 0.1C, decreasing to 573.4, 515.4, and 494.5 mAh g^–1^_(active)_ at 0.5C, 1C, and 2C, and recovering to 642.4 mAh g^–1^_(active)_ at 0.1C (Table S.5). When expressed as retention relative to the initial 0.1C block average, PoSi-10/NGE retains 85.2, 76.6, and 73.5% at 0.5C, 1C, and 2C, respectively, while PoSi-20/NGE retains 77.1, 66.5, and 64.7%. Both electrodes show pronounced reversibility, recovering to 95.5% (PoSi-10/NGE) and 90.8% (PoSi-20/NGE) upon return to 0.1C.

Because these electrodes are composite systems, normalization to total active mass does not directly reflect silicon utilization efficiency, particularly when comparing different silicon loadings. To isolate this effect, the same rate-capability data are additionally expressed as silicon-normalized specific capacity (upper curves in Figure 14). The silicon-normalized capacity was estimated using a theory-weighted partitioning approach commonly applied to silicon-graphite composite anodes,^81^ in which the measured composite capacity is apportioned between silicon and nanographite according to their active-mass fractions and theoretical specific capacities (3579 mAh g^–1^ for Si and 372 mAh g^–1^ for graphite). Under this approximation, the silicon capacity fraction is given by $f_{\mathrm{Si}}=\frac{x_{\mathrm{Si}}Q_{\mathrm{Si}}^{\mathrm{theo}}}{x_{\mathrm{Si}}Q_{\mathrm{Si}}^{\mathrm{theo}}+x_{\mathrm{NG}}Q_{\mathrm{NG}}^{\mathrm{theo}}}$ , where *x*_Si_ and *x*_NG_ are the active-mass fractions of silicon and nanographite, and *Q*_Si_^theo^ (3579 mAh g^–1^) and *Q*_NG_^theo^ (372 mAh g^–1^) are their respective theoretical specific capacities. The silicon-normalized specific capacity is then expressed as *Q*_Si_ = (*f*_Si_ *Q*_meas_)/*x*_Si_, where *Q*_meas_ denotes the measured composite capacity normalized to total active mass.

This treatment assumes that the relative contributions of silicon and nanographite remain approximately constant across the investigated rate range and therefore provides a first-order estimate of silicon utilization rather than a direct deconvolution of intrinsic silicon kinetics.

When evaluated on a per-silicon basis (upper curves in Figure 14), PoSi-10/NGE exhibits consistently higher capacity per gram of silicon than PoSi-20/NGE across all rates, indicating more effective utilization of the silicon fraction at lower silicon loading. Using the rate-step averages, PoSi-10/NGE delivers 3310, 2818, 2534, and 2430 mAh g^–1^_(Si)_ at 0.1C, 0.5C, 1C, and 2C, respectively, with recovery to ∼3157 mAh g^–1^_(Si)_ upon return to 0.1C. In contrast, PoSi-20/NGE delivers approximately 3040, 2343, 2019, and 1964 mAh g^–1^_(Si)_ with recovery to ∼2757 mAh g^–1^_(Si)_. These results reveal a clear trade-off between electrode-level capacity and silicon utilization: increasing silicon content enhances total capacity per gram of active material, whereas lower silicon loading maximizes the capacity extracted per gram of silicon.

Notably, both electrodes exhibit near-complete recovery of capacity when the current density is returned to 0.1C, indicating that the rate-induced losses are largely reversible and not associated with permanent structural degradation. This behavior is consistent with the voltage-profile and differential-capacity analyses discussed earlier. In particular, the persistence of silicon-related delithiation features in the 50th-cycle d*Q*/d*V* profiles, together with the limited polarization growth observed for PoSi-10/NGE, indicates that a substantial fraction of silicon remains electrochemically accessible even under elevated current densities. The achieved pore structure of the PoSi, in combination with the conductive graphite matrix, facilitates lithium-ion transport and supports stable interfacial kinetics at moderate silicon loadings.

Overall, the graphite fraction plays a critical role in the long term capacity retention and mechanical stability of these composites, serving both as an electrochemically active component and as a buffering framework for silicon volume changes. While the incorporation of PoSi clearly extends the reversible capacity and cycling stability of silicon-graphite electrodes, excessive silicon content can outweigh these benefits and lead to rapid performance decay, as observed for PoSi-45/NGE, which converges toward graphite-like behavior after extended cycling. These results underscore the importance of balanced electrode design, in which porosity, silicon loading, and the graphite matrix collectively determine electrochemical durability. In this context, the combined CV, d*Q*/d*V*, and EIS analyses demonstrate that the principal advantage of PoSi at 10–20 wt % lies not only in higher capacity, but in improved interfacial stability under deep cycling, manifested by persistent silicon-related electrochemical signatures and suppressed polarization growth. It should be noted that all electrochemical measurements, including rate capability tests, were conducted at a relatively low areal mass loading of approximately 1 mg cm^–2^, which is below that typically employed in commercial graphite anodes. This loading was intentionally selected to enable clear evaluation of intrinsic material behavior and interfacial kinetics without complications arising from transport limitations or thickness-dependent polarization. While higher mass loadings are expected to introduce additional challenges, the robust rate response and reversible capacity recovery observed here suggest that the porous-silicon design principles identified in this study remain relevant. Future work will therefore focus on translating these concepts to practical, high-loading electrode architectures through increased coating thickness, optimized binder systems, and electrode designs that preserve ionic and electronic percolation at commercially relevant areal capacities.

## 4 Conclusions

This study demonstrates that porous silicon microparticles, synthesized via a green, urea-assisted dual-effect etching process from metallurgical-grade silicon, can effectively stabilize silicon electrochemistry at moderate loadings in lithium-ion battery anodes. Unlike conventional chemical etching methods that require hazardous reagents such as HF, this approach provides a safer and operationally simple route to microparticles exhibiting significantly increased surface area and mesopore contributions, as confirmed by nitrogen sorption analysis. XPS, supported by TGA and structural data, indicates the presence of stabilizing Si–O and Si–N surface species, which correlate with improved interfacial passivation and thermal resistance. When incorporated into composite electrodes with nanographite, these microparticles achieve stable, high reversible capacities: at moderate silicon loadings (10–20 wt %), the electrodes maintain 630–880 mAh g^–1^ for 100 cycles at 0.1 C, exceeding the performance of untreated silicon and pure graphite under identical conditions, while sustaining Coulombic efficiencies above 98.8%. However, higher silicon content results in accelerated capacity fading, highlighting the importance of compositional balance and interfacial stability rather than porosity alone. Taken together, these findings establish urea-assisted porous silicon microparticles as a mechanistically validated platform for silicon-graphite composite anodes and provide a foundation for future studies focused on reagent optimization, higher-loading electrode architectures, and full-cell implementation.

## Supplementary Material



## Notes

High-resolution N 1s XPS spectra of PoSi after thermal treatment at 800 °C; Energy-dispersive X-ray spectroscopy (EDS) elemental mapping images for all prepared electrode formulations; Cyclic voltammograms showing five consecutive cycles for each electrode and Differential capacity (d*Q*/d*V*) analysis; First-cycle cyclic voltammograms, first-discharge differential capacity (d*Q*/d*V*) profiles, evolution of discharge d*Q*/d*V* profiles; Cycling-dependent impedance evolution of composite electrodes measured after the fifth, 20th, and 50th cycles and evolution of *R*_SEI_ and *R*_CT_ extracted from the fits as a function of cycle number; Nitrogen sorption-derived textural properties of PoSi and control samples; Surface atomic composition of pristine silicon and porous silicon estimated from XPS survey spectra; XPS peak positions, assigned species, and relative contributions PrSi, PoSi, and PoSi-800; Equivalent-circuit fitting parameters extracted from electrochemical impedance spectroscopy; Composition and rate-dependent capacity metrics of PoSi-nanographite composite electrodes (PDF)
