# Supplementary material for: Porous Structuring of Si Microparticles for Li-Ion Battery Anodes by Urea-Assisted Etching
Source: ACS Omega. 2026 Feb 16;11(8):13902–21. doi: 10.1021/acsomega.5c12477 (PMC12961504; doi:10.1021/acsomega.5c12477)
Supplement: Supplementary file 1 [file ao5c12477_si_001.pdf]

Supporting information: Porous Structuring of Si Microparticles for Li-Ion Battery Anodes by Urea-Assisted Etching

Ali Abo-Hamad[1], Manisha Phadatare[1], Daniel Brandell[1,2], Maria Hahlin[1,2,3], Jonas Örtegren[1].*

[1] Department of Engineering, Mathematics and Science Education (IMD), Mid Sweden University, Sundsvall, SE-851 70, Sweden

[2] Department of Chemistry - Ångström Laboratory, Uppsala University, Uppsala, SE-751 21, Sweden

[3] Department of Physics and Astronomy; X-ray Photon Science, Uppsala University, Uppsala, SE-752 37 UPPSALA, Sweden

[*] Corresponding author:

Jonas Örtegren:

E-mail addresses: jonas.ortegren@miun.se

Address: Department of Engineering, Mathematics and Science Education (IMD), Mid Sweden University, Sundsvall, SE-851 70, Sweden

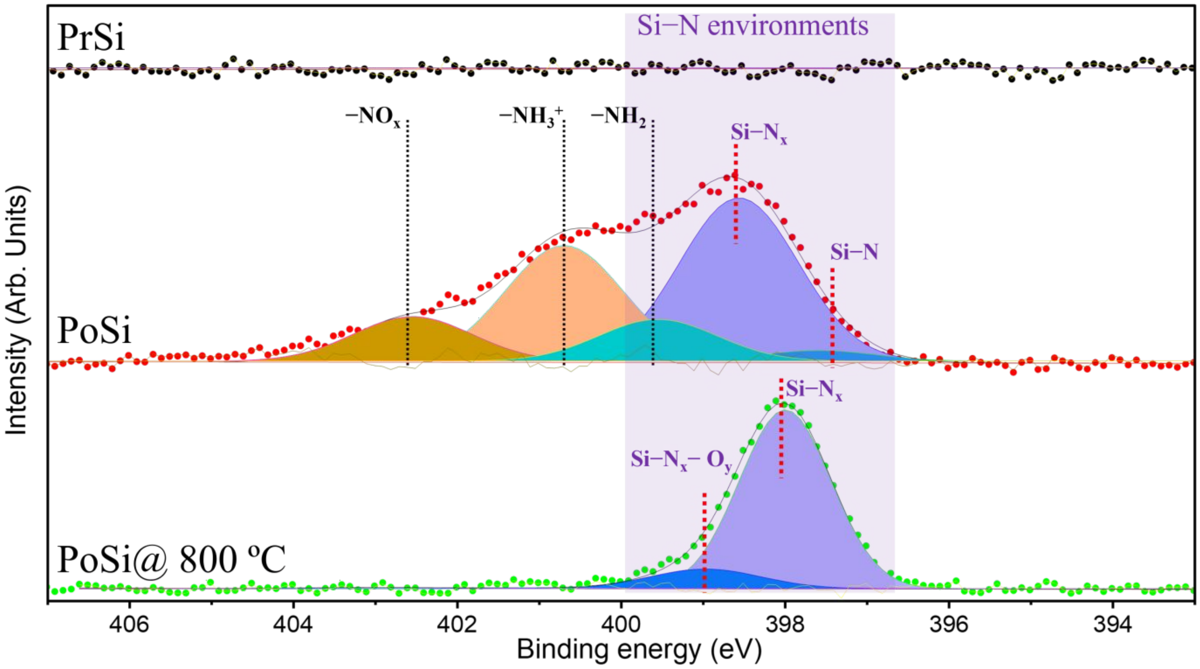

Figure S. 1. High-resolution N 1s XPS spectra of PoSi after thermal treatment at 800 ˚C, showing deconvoluted Si–N–related components and fitted envelope.

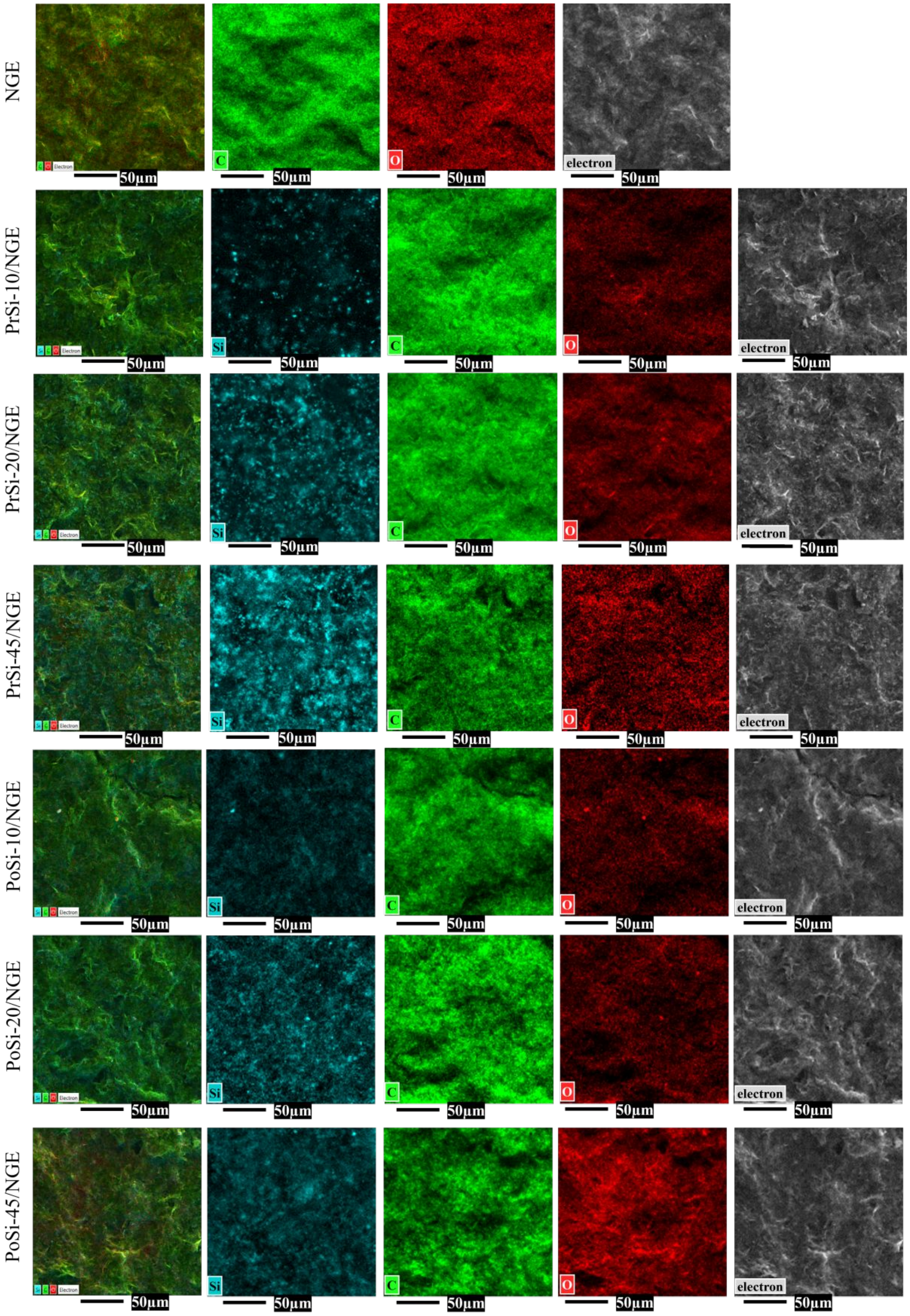

NGE
PrSi-10/NGE
PrSi-20/NGE
PrSi-45/NGE
PoSi-10/NGE
PoSi-20/NGE
PoSi-45/NGE
Si
C
O
electron
50μm

Figure S. 2. EDS elemental mapping of freshly prepared electrodes (see Table 1) showing spatial distribution of silicon (Si), carbon (C), and oxygen (O), alongside corresponding electron images.

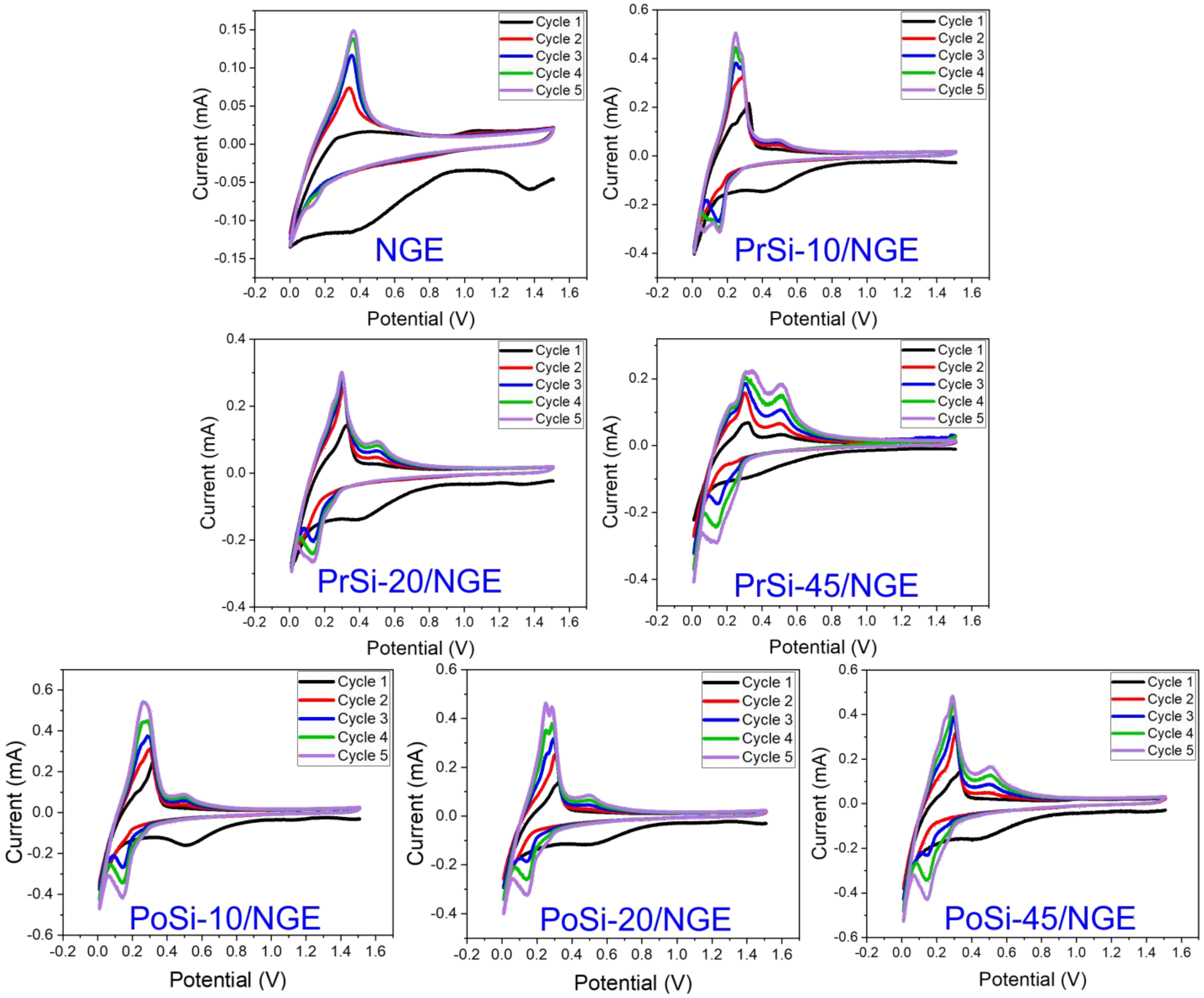

Figure S. 3. Cyclic voltammograms (five consecutive cycles) for graphite-based electrode (NGE) and composite electrodes (PrSi/NGEs and PoSi/NGEs).

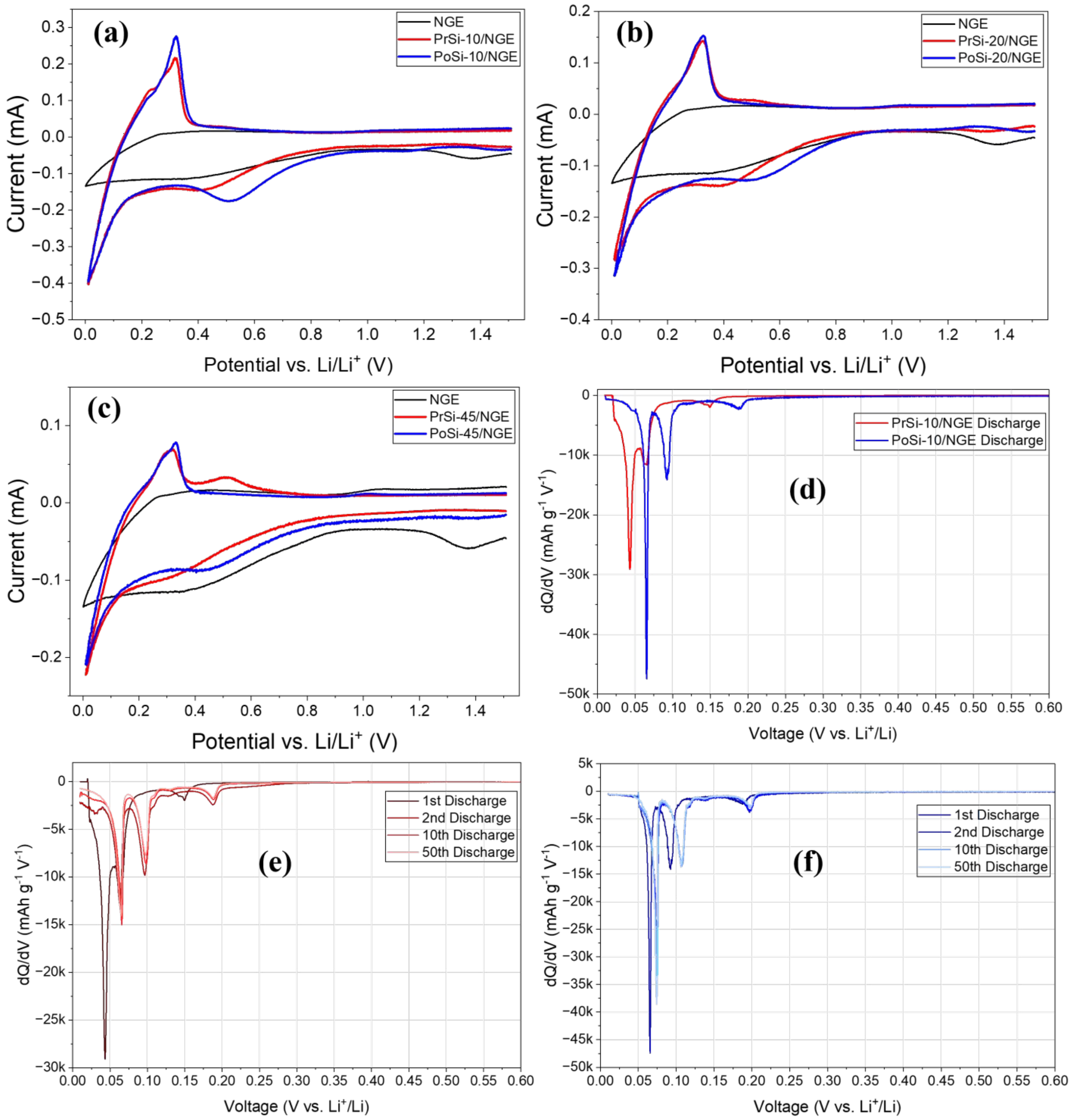

Figure S. 4. (a–c) First-cycle cyclic voltammograms of NGE, PrSi/NGEs, and PoSi/NGEs at silicon loadings of 10, 20, and 45 wt%, highlighting differences in initial cathodic reduction behavior. (d) First-discharge differential capacity (dQ/dV) profiles of PrSi-10/NGE and PoSi-10/NGE derived from galvanostatic cycling. (e,f) Evolution of discharge dQ/dV profiles for

PrSi-10/NGE and PoSi-10/NGE at the 1st, 2nd, 10th, and 50th cycles. All dQ/dV values are normalized to the mass of active material.

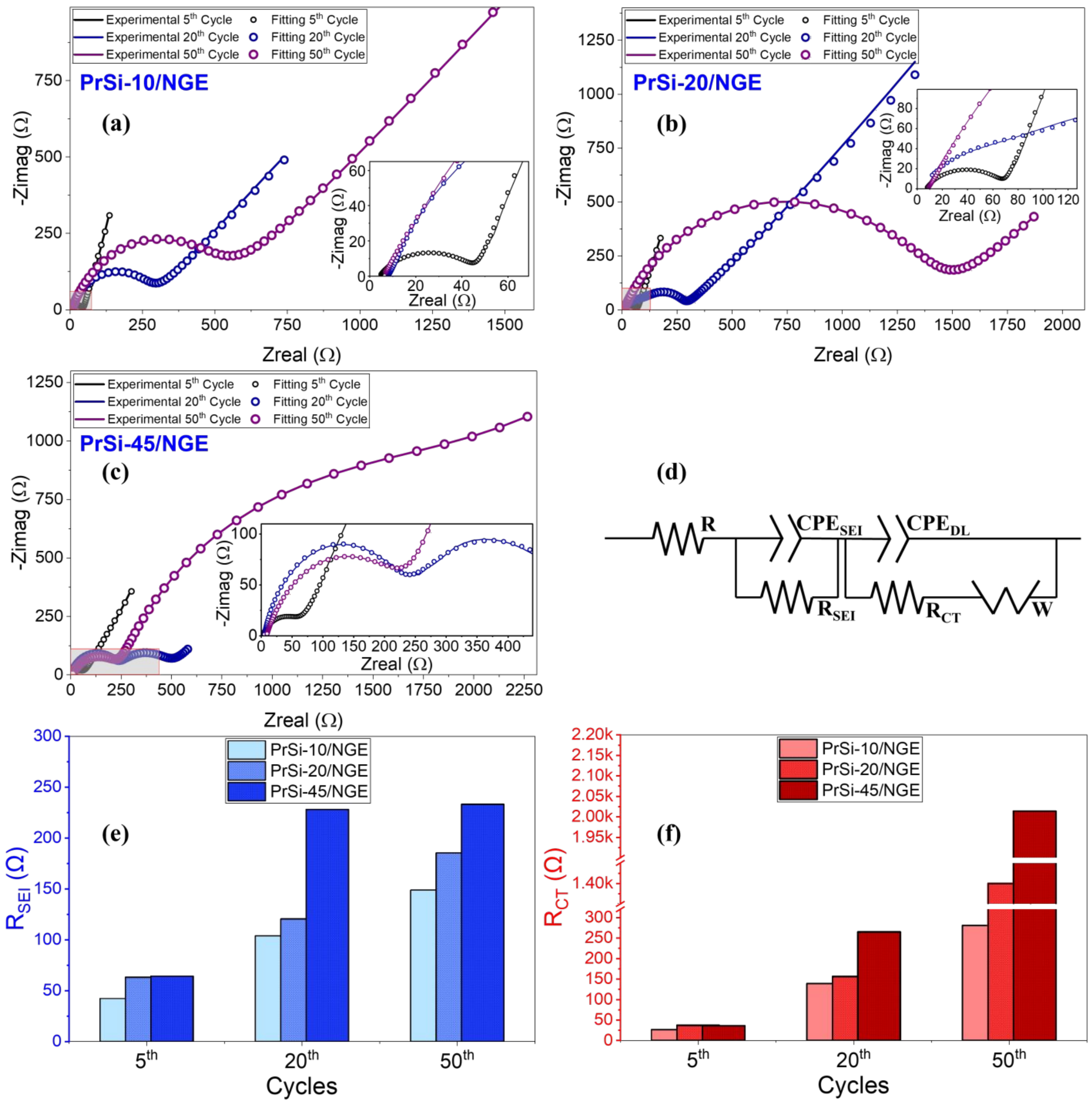

Figure S. 5. Cycling-dependent impedance evolution of pristine-silicon composite electrodes. (a-c) Nyquist plots of PrSi-10/NGE, PrSi-20/NGE, and PrSi-45/NGE measured after the 5th, 20th,

and 50th cycles (solid lines: experimental data; symbols: fits). (d) Equivalent circuit used for fitting. (e) Evolution of R_{SEI} and (f) R_{CT} extracted from the fits as a function of cycle number.

Table S. 1. Nitrogen sorption-derived textural properties of PoSi and control samples.

Sample	BET surface area (m^2 g^{-1})	Highest measured (p/p^0)	N_2 adsorbed at p/p^0_{max} (mmol g^{-1})	Total pore volume, V_{total} (cm^3 g^{-1})	V_{meso} (2-50 nm, BJH) (cm^3 g^{-1})	Dominant BJH peak (nm)	Secondary BJH feature (nm)
**Control 1 (Only thermal)**	2.6 ± 0.02	0.9956	0.6043	0.0209	~0.00001	~2 (weak)	–
**Control 2 (Minimum chemical)**	4.2 ± 0.03	0.9836	0.8677	0.0301	0.01042	~4 (minor)	~30-40
**Control 3 (Minimum mechanical)**	7.2 ± 0.02	0.9892	1.2191	0.0422	0.01627	~4 (dominant)	–
**PoSi (Dual effect)**	26.6 ± 0.02	0.9851	1.7293	0.0599	0.06365	~4 (dominant)	~30-40

Total pore volume was calculated from nitrogen uptake at the highest measured relative pressure p/p^0_{max}. BJH mesopore volume (V_{meso}) was obtained by trapezoidal integration of BJH pore-size distributions (dV/dlog(w)) over the 2-50 nm range. BJH values represent the contribution of BJH-resolved mesopores and are not cumulative pore volumes.

Table S. 2. Surface atomic composition (atomic %) of pristine silicon (PrSi) and porous silicon (PoSi) estimated from XPS survey spectra using background-subtracted peak areas and published sensitivity factors.

**Sample**	**Atomic Contribution %**			
	**Si (Si 2p)**	**C (C 1s)**	**N (N 1s)**	**O (O 1s)**
**PrSi**	33.1	24.5	ND	42.4
**PoSi**	33.4	5.2	2.6	58.8

Surface elemental compositions were estimated from XPS survey (wide-scan) spectra by integrating background-subtracted peak areas corresponding to the Si 2p, C 1s, N 1s, and O 1s core levels. Atomic concentrations were calculated using relative sensitivity factors (RSFs) for Al Kα excitation taken from published average-matrix relative sensitivity factor (AMRSF) tables, with C 1s used as the reference (C 1s = 1). For Si, the total Si 2p envelope was considered as the sum of the $2p_{3/2}$ and $2p_{1/2}$ contributions. Atomic percentages were obtained according to:

$$Atomic\%_i = 100 \times \frac{A_i/RSF_i}{\sum_j (A_j/RSF_j)}$$

where A_{i} is the integrated peak area of element i after background subtraction, and the summation j runs over all elements detected in the survey spectrum Nitrogen was not detected in the pristine silicon (PrSi) sample, as no discernible N 1s signal above the noise level was observed, and is therefore reported as not detected (ND). It should be noted that XPS-derived atomic concentrations are semi-quantitative and sensitive to the choice of background subtraction, integration limits, and sensitivity factors, as well as to surface roughness and

chemical inhomogeneity, particularly for porous materials. Consequently, the reported atomic percentages should be regarded as estimates of near-surface composition rather than absolute stoichiometric values.[1,2]

Table S. 3. XPS peak positions, assigned species, and relative contributions (%) for Si 2p, C 1s, N 1s, and O 1s core levels of PrSi, PoSi, and PoSi-800.

Core level	Sample	Peak position (eV)	Assigned species	Contribution (%)
Si2p	PrSi	~98.8-99.5	S^0	36.4
		~100.1-100.5	Si^{1+}	13.5
		~101.1	Si^{2+}	6.3
		~102.5-103.1	Si^{3+}	3.6
		~103.4–105.6	Si^{4+}	40.2
	PoSi	~98.8-99.4	S^0	0.4
		~100.4-101.0	Si^{1+}	0.8
		~102.4-103.0	Si^{2+}	4.6
		~103.5-107.0	Si^{4+}	94.2
C1s	PrSi	~285.2	C–C / C–H	55.5
		~286.5	C–O	25.1
		~287.5	C=O	6.5
		~289.1	O–C=O	12.9
	PoSi	~284.7	C–C / C–H	46
		~286.0	C–O	19.5
		~287.2	C=O	19.5
		~289.0	O–C=O	15.0
N1s	PrSi	None	-	-
	PoSi	~397.6	Si–N	2.6
		~398.5	$Si–N_x$	43.5
		~399.5	$–NH_2$	11.1
		~400.7	$–NH_3^+$	30.9
		~402.5	NO_x	11.9
	PoSi-800	~398.0	$Si–N_x$	88.2
		~399.0	$Si-N_x-O_y$	11.8
O1s	PrSi	~528.5	Si–O lattice-like O in SiO_x	73.8
		~530.3	Si–O defect oxide SiO_x	24.7
		~529.9	hydroxyl/adsorbed O	1.5
	PoSi	~532.8	Si–O	53.2

	~534.6	–OH / carbonate/ adsorbed O	46.2
	~534.2	adsorbed O species	0.6

Table S. 4. Equivalent-circuit fitting parameters extracted from electrochemical impedance spectroscopy (EIS) measurements of composite electrodes after five CV cycles.

Electrode	R (Ω)	R_{SEI} (Ω)	R_{CT} (Ω)
PrSi-10/NGE	4.4	42.1	26.1
PoSi-10/NGE	9.8	56.1	31.5
PrSi-20/NGE	7.0	63.1	37.1
PoSi-20/NGE	8.1	81.2	44.6
PrSi-45/NGE	1.8	64.1	35.9
PoSi-45/NGE	9.4	94.1	79.4

Table S. 5. Composition and rate-dependent capacity metrics of PoSi-nanographite composite electrodes.

Electrode Properties	PoSi-10/NGE	PoSi-20/NGE
Active composition (Si:NG)	1 : 8	2 : 7
Active mass fraction, x_{Si}	0.11	0.22
Active mass fraction, x_{NG}	0.89	0.78
Theoretical electrode capacity (per g $_{active}$)	728 mAh g^{-1}	1084 mAh g^{-1}
Capacity contribution from Si	54.6%	73.3%
Capacity contribution from NG	45.4%	26.7%
Avg. capacity at 0.1C (cycles 1-10, per g $_{active}$)	672.8 mAh g^{-1} (100% Retention)	920.4 mAh g^{-1} (100% Retention)
Avg. capacity at 0.5C (cycles 11-20, per g $_{active}$)	573.4 mAh g^{-1} (85.2% Retention)	709.8 mAh g^{-1} (77.1% Retention)
Avg. capacity at 1C (cycles 21-30, per g $_{active}$)	515.4 mAh g^{-1} (76.6% Retention)	612.0 mAh g^{-1} (66.5% Retention)
Avg. capacity at 2C (cycles 31-40, per g $_{active}$)	494.5 mAh g^{-1} (73.5% Retention)	595.2 mAh g^{-1} (64.7% Retention)
Avg. capacity at 0.1C (recovery cycles 41-50, per g $_{active}$)	642.4 mAh g^{-1} (95.5% Retention)	835.3 mAh g^{-1} (90.8% Retention)

References

(1) Chastain, J.; King Jr, R. C. Handbook of X-Ray Photoelectron Spectroscopy. *Perkin-Elmer Corp.* **1992**, *40* (221), 25.
(2) Cant, D. J. H.; Gorham, J. M.; Clifford, C. A.; Shard, A. G. Standard Approaches to XPS and AES Quantification—A Summary of ISO 18118:2024 on the Use of Relative Sensitivity Factors. *Surf. Interface Anal.* **2025**, *57* (2), 148–152. https://doi.org/10.1002/sia.7371.
